# Improved hyperacuity estimation of spike timing from calcium imaging

**DOI:** 10.1038/s41598-020-74672-y

**Published:** 2020-10-20

**Authors:** Huu Hoang, Masa-aki Sato, Shigeru Shinomoto, Shinichiro Tsutsumi, Miki Hashizume, Tomoe Ishikawa, Masanobu Kano, Yuji Ikegaya, Kazuo Kitamura, Mitsuo Kawato, Keisuke Toyama

**Affiliations:** 1 ATR Brain Information Communication Research Laboratory Group, Advanced Telecommunications Research Institute International, Kyoto, Japan; 2 Department of Physics, Kyoto University, Kyoto, Japan; 3 Laboratory for Multi-scale Biological Psychiatry, RIKEN Center for Brain Science, 2-1 Hirosawa, Wako city, 351-0198 Saitama Japan; 4 Department of Biochemistry, Faculty of Medicine, Saitama Medical University, Saitama, Japan; 5 Graduate School of Pharmaceutical Sciences, The University of Tokyo, Tokyo, Japan; 6 Department of Neurophysiology, Graduate School of Medicine, The University of Tokyo, Tokyo, Japan; 7 Department of Neurophysiology, Faculty of Medicine, University of Yamanashi, Yamanashi, Japan

**Keywords:** Computational neuroscience, Ca2+ imaging

## Abstract

Two-photon imaging is a major recording technique used in neuroscience. However, it suffers from several limitations, including a low sampling rate, the nonlinearity of calcium responses, the slow dynamics of calcium dyes and a low SNR, all of which severely limit the potential of two-photon imaging to elucidate neuronal dynamics with high temporal resolution. We developed a hyperacuity algorithm (HA_time) based on an approach that combines a generative model and machine learning to improve spike detection and the precision of spike time inference. Bayesian inference was performed to estimate the calcium spike model, assuming constant spike shape and size. A support vector machine using this information and a jittering method maximizing the likelihood of estimated spike times enhanced spike time estimation precision approximately fourfold (range, 2–7; mean, 3.5–4.0; 2SEM, 0.1–0.25) compared to the sampling interval. Benchmark scores of HA_time for biological data from three different brain regions were among the best of the benchmark algorithms. Simulation of broader data conditions indicated that our algorithm performed better than others with high firing rate conditions. Furthermore, HA_time exhibited comparable performance for conditions with and without ground truths. Thus HA_time is a useful tool for spike reconstruction from two-photon imaging.

## Introduction

Recently, because of its high spatial resolution, two-photon imaging has been one of the major means of recording multi-neuronal activities in the field of neuroscience to determine the precise morphology and location of the target neurons^1–6^. However, it has relatively low temporal resolution due to the mechanical scanning of two-photon rays, which limits its utility. Other problems include nonlinearity, slow dynamics, and a low signal-to-noise ratio (SNR) of calcium (Ca) responses^7–10^. To overcome these problems, many algorithms for reconstructing spike trains from Ca imaging data have been proposed, including conventional thresholding^11^, deconvolution^12–15^, template matching^16–20^, Bayes inference^21–23^, and machine learning^24,25^. However, few of these proposed algorithms have simultaneously addressed the two challenging goals: (1) reliable spike detection and (2) spike time estimation with high temporal precision in the presence of nonlinearity, slow dynamics and low SNR of the Ca responses^26^. It is important to achieve both goals because recent neuroscience has indicated that the information provided by temporal coding based on neuronal spikes is as important as that provided by spike rate coding^27,28^.

Regarding the former goal, the spike dynamics of the target neurons and/or the kinematics of the Ca responses may vary dramatically across brain regions and Ca dyes. Regarding the latter goal, there is a trade-off between the number of recorded neurons and temporal resolution. In addition, the slow kinematics and low SNR of currently available Ca dyes may limit the temporal precision of the information conveyed by Ca responses. These factors hinder reliable spike detection and precise spike time estimation for high-frequency firing, which is common in cortical cells^29–31^.

In this paper, we propose a hyperacuity algorithm, named HA_time (HyperAcuity time estimation), that combines a generative model of Ca responses, including nonlinearity and dye dynamics, with a supervised classifier to overcome the aforementioned difficulties. HA_time estimates the Ca spike model by Bayesian inference, assuming a constant size and shape; compensates for the nonlinearity of Ca responses; and detects spikes from Ca imaging data by a support vector machine (SVM) using the ground truths (i.e., simultaneously recorded electrical spikes) as supervised information. To achieve hyperacuity precision^32^, spike timings were calibrated to minimize the residual errors in model prediction, using the hyperacuity vernier scale tenfold finer than the sampling interval. This approach benefits from the advantages of both generative models and supervised learning. The Ca spike model is utilized to provide supplemental information for spike detection and to estimate spike times with higher temporal precision than the sampling resolution. Supervised learning compensates for fluctuations in the Ca responses that are due to noise and sampling jitters, which are not considered by the generative model. As a consequence, HA_time can improve both the spike detection and spike time estimation of two-photon recordings.

A simulation study proved that HA_time obtained an approximately fourfold improvement (range, 2–7; mean, 3.5–4.0; 2SEM, 0.1–0.25) over the sampling rate of 10–60 Hz, even when the Ca dye kinematics and noise level varied as in the experimental conditions. All algorithms aiming to improve spike detection as well as spike time precision have used generative models and maximized the likelihood of estimates ^17,22,23^. Accordingly, hyperacuity improvement was limited to only the cases in which the Ca responses satisfied the assumptions of generative models. To prove the advantages of an approach that combines a generative model and supervised learning, we compared our method with four previously introduced hyperacuity algorithms^16,17,21,22^ and one representative deconvolution algorithm not aiming for hyperacuity^15^. The benchmark results for the three biological data sets (visual cortex, hippocampus, and cerebellum) showed that HA_time was among the best algorithms, with statistically improved hyperacuity demonstrated for the visual cortex and hippocampus data, but not for the cerebellum data, presumably due to the small number of samples. The simulation analysis, which was conducted across a broad range of parameters for the experimental conditions (e.g., mean neuronal firing frequency, nonlinearity, decay time of Ca dyes, noise level, sampling rate), provided useful information for users aiming to select the most suitable algorithms for a given experimental condition. The superiority of our algorithm was especially evident in cases of high firing frequency and/or strong nonlinearity, which are common in the cortical cells of behaving animals.

The advantage to combine generative model with the supervised learning was also evident for unsupervised version of HA_time. In this unsupervised algorithm, SVM was trained with the simulation data produced by the generative model (hyperacuity Bayes). It exhibited comparable performances for the experimental data with no ground truths to those with the ground truths. Therefore, supervised and unsupervised HA_time may be useful for cases with and without the ground truths, respectively.

## Results

### Hyperacuity algorithm for spike timing estimation

HA_time was implemented in three steps: (1) Bayesian inference of the Ca spike model from Ca imaging data; (2) spike detection by SVM assisted by the matching information of Ca imaging data and Ca spike model; and (3) hyperacuity spike time estimation using the hyperacuity vernier scale to minimize the errors between the Ca response model prediction and the Ca imaging data. Here, the term “Ca spike model” refers to the constant Ca transient (i.e., the amplitude and shape) of a single spike, whereas “Ca response model” refers to a generative model of the Ca spike model, superposition of multiple spikes, nonlinearity of the Ca responses, and noise.

**Figure 1:**
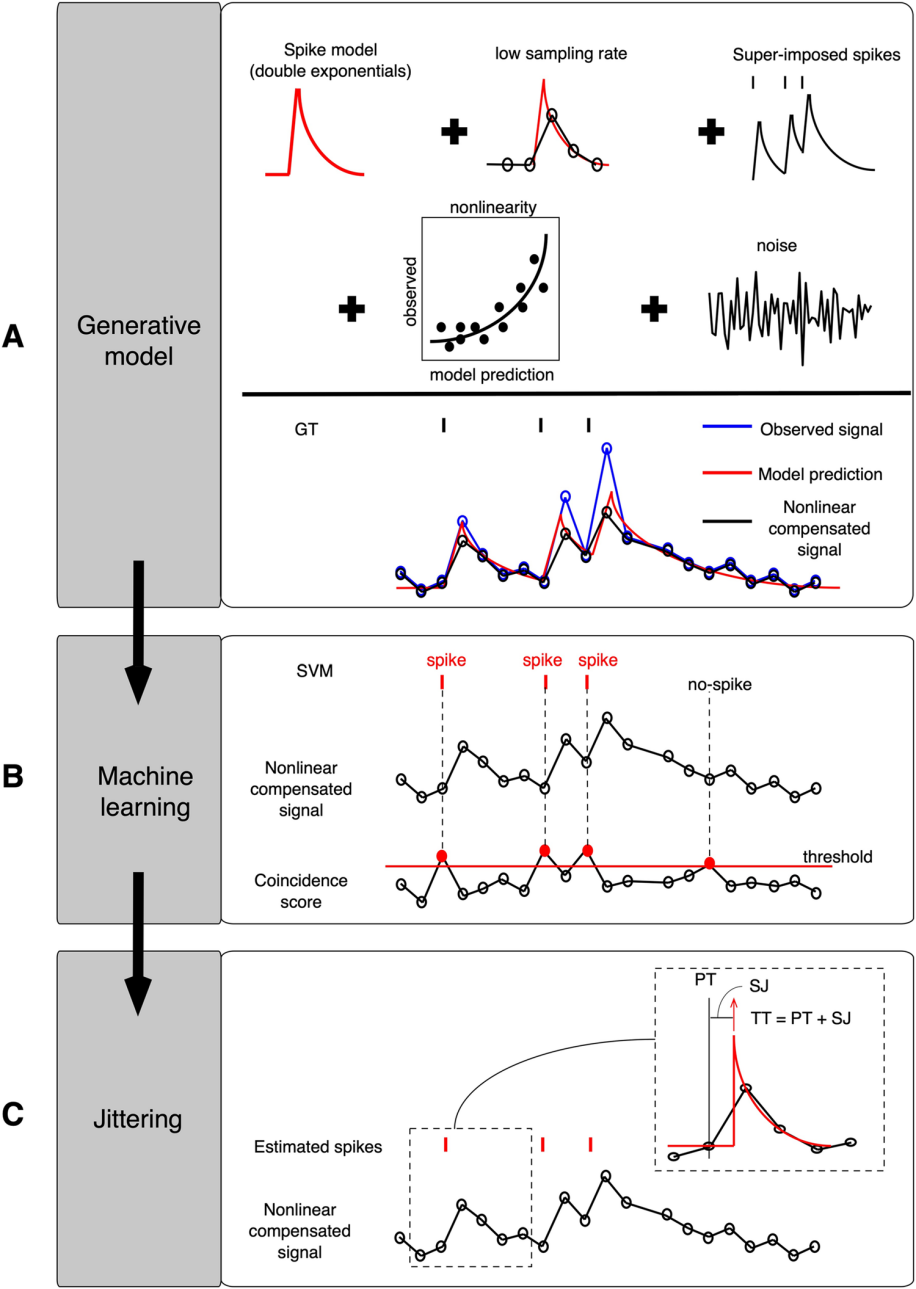
The hyperacuity support vector machine (HA_time) algorithm. (**A**) The generative model assumed that Ca responses were sampled from the Ca spike model (double exponentials) with variable sampling jitters (SJs) due to the low sampling rate. The model featured multiple superimposed spikes, fluctuating nonlinearity, and supplemented Gaussian noise. The nonlinearity observed in the data was compensated for by the nonlinearity model, which was defined by the logarithmic functions of the observed Ca imaging data and Ca response model prediction. (**B**) The coincidence score, which was computed by convoluting the first derivatives of the Ca imaging data and the Ca spike model, was used to select spike candidates. An SVM was trained to classify spikes and non-spikes from a set of spike candidates using the Ca imaging data, coincidence scores, and electrical spikes as feature, attribute, and teaching signals, respectively. (**C**) The true spike time (TT) was estimated as the sum of the SJ and pseudo-spike time (PT, the point that exceeds the threshold), minimizing the residual error of the Ca response model prediction using a hyperacuity vernier scale 10 times finer than the sampling interval.

We assumed that the Ca imaging data were sampled from the Ca spike model (double exponentials) with variable sampling jitters (SJs) between the onsets of the Ca spike and the sampling times. First, the Ca spike responses were linearly superimposed for multiple spikes in short intervals and then added with the Gaussian noise. Then, the sublinearity or superlinearity of the Ca responses was determined by comparing the observed Ca imaging data with the data predicted by the Ca response model. We compensated for nonlinearity by inversely transforming the observed Ca imaging data using nonlinearity models fitted by logarithmic functions (Fig. 1A).

Next, we estimated the coincidence score as a convolution of the first-order derivatives of the Ca imaging data and the Ca spike model. A coincidence score threshold was used to sample the data segments as spike candidates, and the threshold and segment size were optimized to maximize the F1 score of the training data (see “Methods”). The SVM was trained to classify the sampled data segments into spike or non-spike segments. For this purpose, we fed the sampled Ca imaging data and the coincidence scores to the SVM as the primary and attribute inputs, respectively, and used the electrical spikes (ground truth) as the teaching signals (Fig. 1B).

For the test data, spike candidates were sampled in the same way as the training data, and the trained SVM detected spikes among the candidates. We tentatively determined the time when the coincidence score exceeded the threshold as the pseudo-spike time (PT; Fig. 1C) and estimated the SJs to minimize the errors between the Ca response model prediction and the Ca imaging data using the hyperacuity vernier scales (with a time bin 10 times finer than the sampling interval). The true spike time (TT) was calculated as the sum of the PT and SJ (Fig. 1C).

The three steps of HA_time described above were crucial for estimating the hyperacuity spike timing from the Ca imaging data. The generative model worked as a forward model to transform spiking activities into Ca signals and to resolve the inverse problem of reconstructing spike activities from the Ca signals by estimating the model parameters. The SVM worked as a signal classifier to enhance the precision of spike detection by selecting spikes from the spike candidates, assisted by matching information of the Ca signals and the Ca spike model. Finally, the precision of spike time was enhanced by jitter processing to achieve hyperacuity.

### Hyperacuity improvement of HA_time in a simulation

We estimated the hyperacuity index (sampling interval/spike time error) for hit cases of simulation data (see Methods) and compared performance with OASIS^15^, as a representative deconvolution algorithm aiming at no hyperacuity.

**Figure 2:**
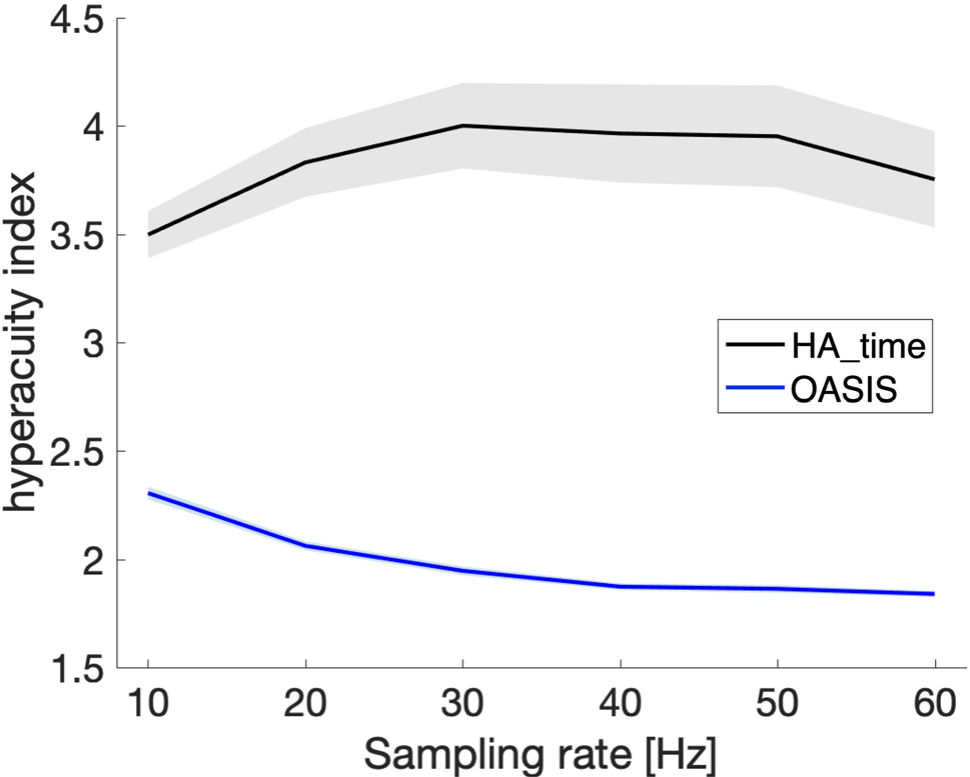
Hyperacuity index of HA_time and OASIS for the simulation data. Ordinate: hyperacuity index for HA_time (black line) and OASIS (blue line). Abscissa: sampling rate of simulation data with a mean neuronal firing rate of 1 Hz. The shaded region indicates ± 2SEM when the nonlinearity parameter (α), decay time constant (τ_2_), and SNR were within the ranges of 0.5–1.5, 0.2–1 s, and 3–10, respectively.

Figure 2 plots the hyperacuity index as a function of the sampling rate. The hyperacuity index of HA_time was maintained at around 4 (range, 2–7; mean, 3.5–4.0, 2SEM, 0.1–0.25) across the entire range of sampling rates (10–60 Hz), even when other parameters varied, as in the experimental conditions. As expected, the hyperacuity index of OASIS remained around 2, which is the theoretical (Nyquist) limit of temporal precision for digital sampling.

### Application of HA_time to the experimental data

We applied HA_time to the noisy Ca imaging data obtained from cerebellar, hippocampal, and primary visual cortical cells by two-photon recording with a relatively low sampling rate.

**Figure 3:**
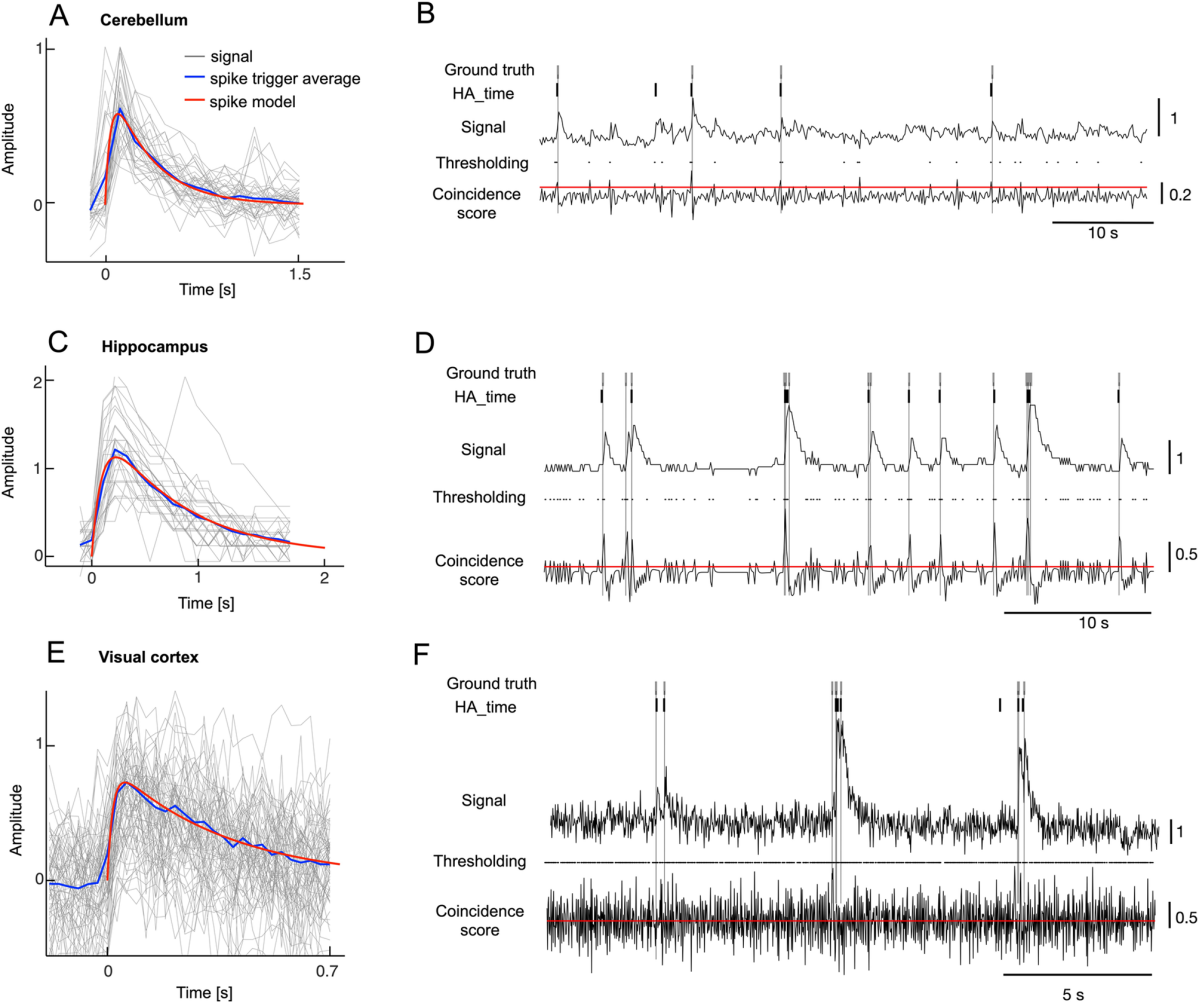
Estimation of the Ca spike model and spike detection by HA_time. (**A**) Ca spike model (red line, τ_1_, 0.05 s, τ_2_, 0.4 s) and average spike-triggered Ca responses synchronized with the onsets of electrical spikes. The Ca spike model and the spike-triggered average were estimated for the 36 electrical spikes of five Purkinje cells. Ordinate: amplitude of Ca responses normalized by the peak of the maximum Ca imaging data for the individual cells. Abscissa: time after the onset of the electrical spikes. (**B**) Spike detection by HA_time. The top and bottom traces represent Ca imaging data and the coincidence score of the first-order differential of the Ca imaging data and that of the Ca response model. The candidate spikes detected by conventional thresholding, those estimated by HA_time, and electrical spikes (ground truth) are represented by black dots and thick and thin bars, respectively. Red lines indicate the thresholds for conventional thresholding. (**C**, **D**, **E** and **F**) are similar to A and B but represent the hippocampus (**C**, **D**) using OGB-1AM dye and visual cortex data (**E**, **F**) using GCaMP6f dye (see Table S1).

For the two-photon recording of five Purkinje cells in the cerebellum (Cal-520 dye; sampling rate, 7.8 Hz), we sampled 36 data segments (segmental length, 2 s), each of which included a single electrical spike from the simultaneous electrical recording (sampling rate, 20 kHz), and constructed the Ca spike model using Bayesian inference. In agreement with the assumption of a constant spike shape, the Ca spike model (τ_1_ = 0.05 s, τ_2_ = 0.4 s) was slightly faster in rise time (red line in Fig. 3A) than the electrical spike-triggered average of the Ca imaging data (blue line in Fig. 3A). In addition, the amplitude of the Ca spike model roughly corresponded to that of the spike-triggered average. The longer time course of the spike-triggered response may be due to the SJs.

We performed a similar estimation of the Ca spike models for the data from the hippocampus (n = 9 cells, OGB-1AM dye, sampling rate of 10 Hz) and visual cortex (n = 29 cells, three different GCaMP dyes, sampling rate of 50–60 Hz, see Table S1). We avoided segments that contained burst activity (inter-spike interval, < 2 s) because the Ca responses in the hippocampus and visual cortex showed strong nonlinearity during burst activity. A similar tendency, specifically, the rise time being faster than spike-triggered averages (blue lines in Fig. 3C,E), was also noticed in the Ca spike model of data from both the hippocampus (OGB-1AM dye, τ_1_ = 0.1 s, τ_2_ = 0.75 s, red line in Fig. 3C) and visual cortex (GCaMP6f dye, τ_1_ = 0.01 s, τ_2_ = 0.2 s, red line in Fig. 3E). The dynamics of the other two Ca dyes, GCaMP6s and GCaMP5k, for the visual cortex, as well as those for the cerebellum and hippocampus, estimated by HA_time were all in good agreement with those reported in the previous studies^11,33–35^ (see Table S1).

Figure 3B illustrates the performance of HA_time for detecting spikes from the Ca imaging data of cerebellar cortex cells. Coincidence thresholding (black dots in Fig. 3B) detected the true spikes (ground truth, gray bars) as well as many false positive spikes. HA_time effectively selected the true spikes, rejecting many false positive spikes from the set of candidates. A comparison of the spikes detected by HA_time (dark bars) with the ground truths (gray bars) indicated that HA_time detected nearly all spikes and correctly estimated the spike time from the Ca imaging data for the cerebellum (Fig. 3B). Similarly, HA_time estimated the spike times very close to the actual times for the hippocampus and visual cortex data (Fig. 3D,F).

### Nonlinearity analysis of the experimental data sets

We found strong nonlinearity in the Ca imaging data from the hippocampus and visual cortex during burst activities. Therefore, a nonlinearity analysis was conducted by plotting the amplitudes of the Ca imaging data as a function of the linear prediction of the Ca response model for all of the data from the cerebellum, hippocampus and visual cortex.

**Figure 4:**
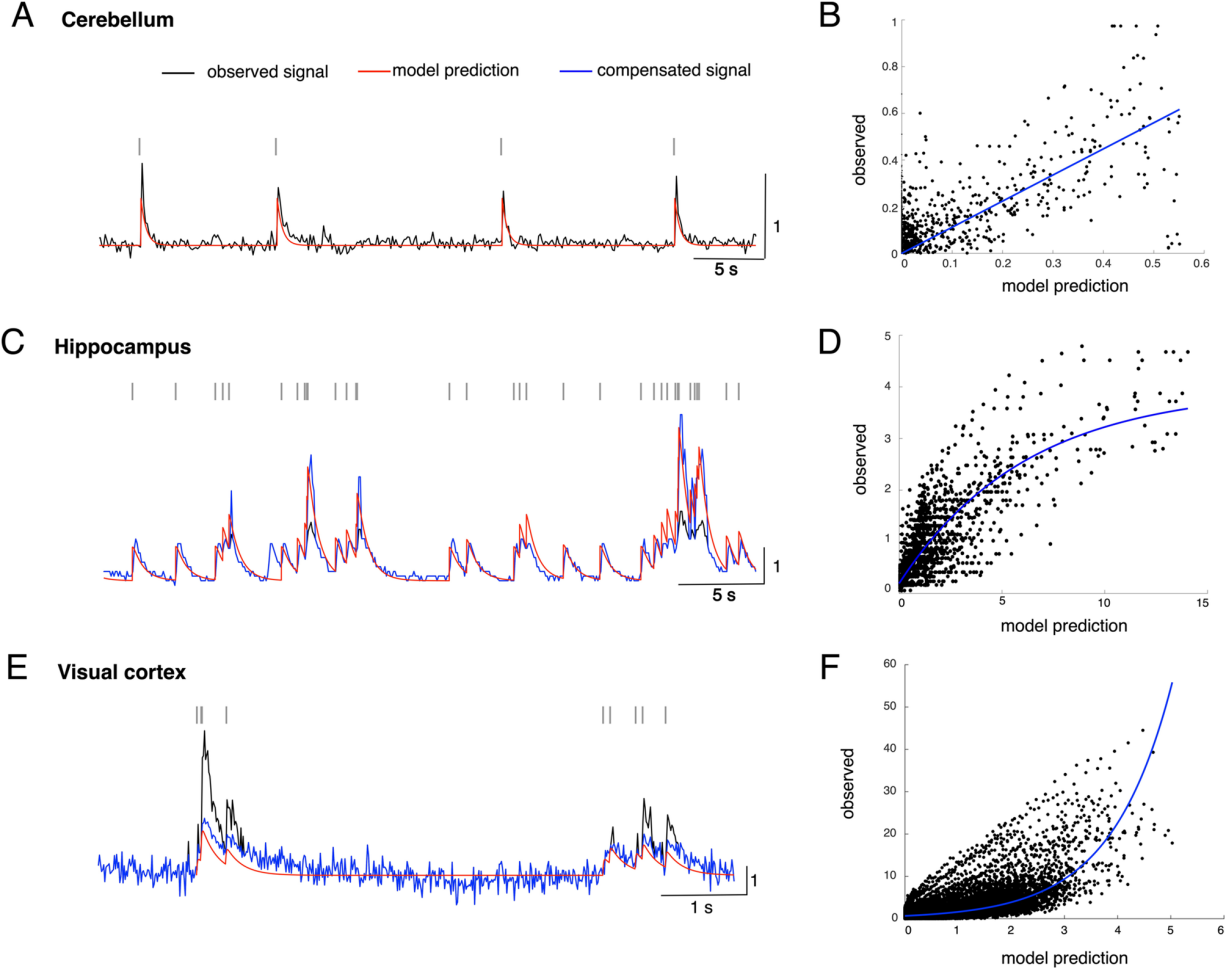
Nonlinearity analysis of Ca imaging data. Ca imaging data from the cerebellum (**A**), hippocampus (**C**) and visual cortex (**E**). Black, red, and blue traces represent the observed Ca imaging data, linear prediction of the Ca response model for spike trains, and compensated Ca imaging data, respectively. Scatter plots of the experimental Ca imaging data from the cerebellum (**B**), hippocampus (**D**) and visual cortex using the GCaMP6f dye (**F**) as a function of the linear prediction of the Ca response model for spike trains.

The Ca imaging data from the cerebellum roughly agreed with the linear prediction for spike trains (Fig. 4A, black and red lines), and correspondingly, the regression analysis revealed a fine match between the two (blue line in Fig. 4B, y = *1.1 x*). Conversely, the nonlinearity analysis of the Ca imaging data revealed significant sublinearity in the hippocampus (OGB-1AM dye, black and red lines in Fig. 4C) and superlinearity in the visual cortex (GCaMP6f dye, black and red lines in Fig. 4E). The nonlinearity models were constructed by fitting the plots with logarithmic functions (blue lines in Fig. 4D,F, y = *-3.8 e*^*(-0.16x*^^*)*^ + *3.9* and *y* = *0.67 e*^*0.88x*^). We found that the Ca imaging data recorded using the GCaMP6s and GCaMP5k dyes for the visual cortex showed the same tendency for superlinearity as when the GCaMP6f dye was used (Fig. S1). The nonlinearity in the hippocampus and visual cortex data was compensated for by multiplying the Ca imaging data by the inverse of the nonlinearity models (blue lines in Fig. 4C,E). The compensated Ca imaging data were then fed into HA_time.

### Performance evaluation for experimental data

The performances of HA_time for detecting spikes and estimating the spike time were examined for the cerebellum, hippocampus, and visual cortex data by leave-one-out cross-validation and then compared with the performance of five benchmark algorithms^15–17,21,22^ (see “Methods”).

**Figure 5:**
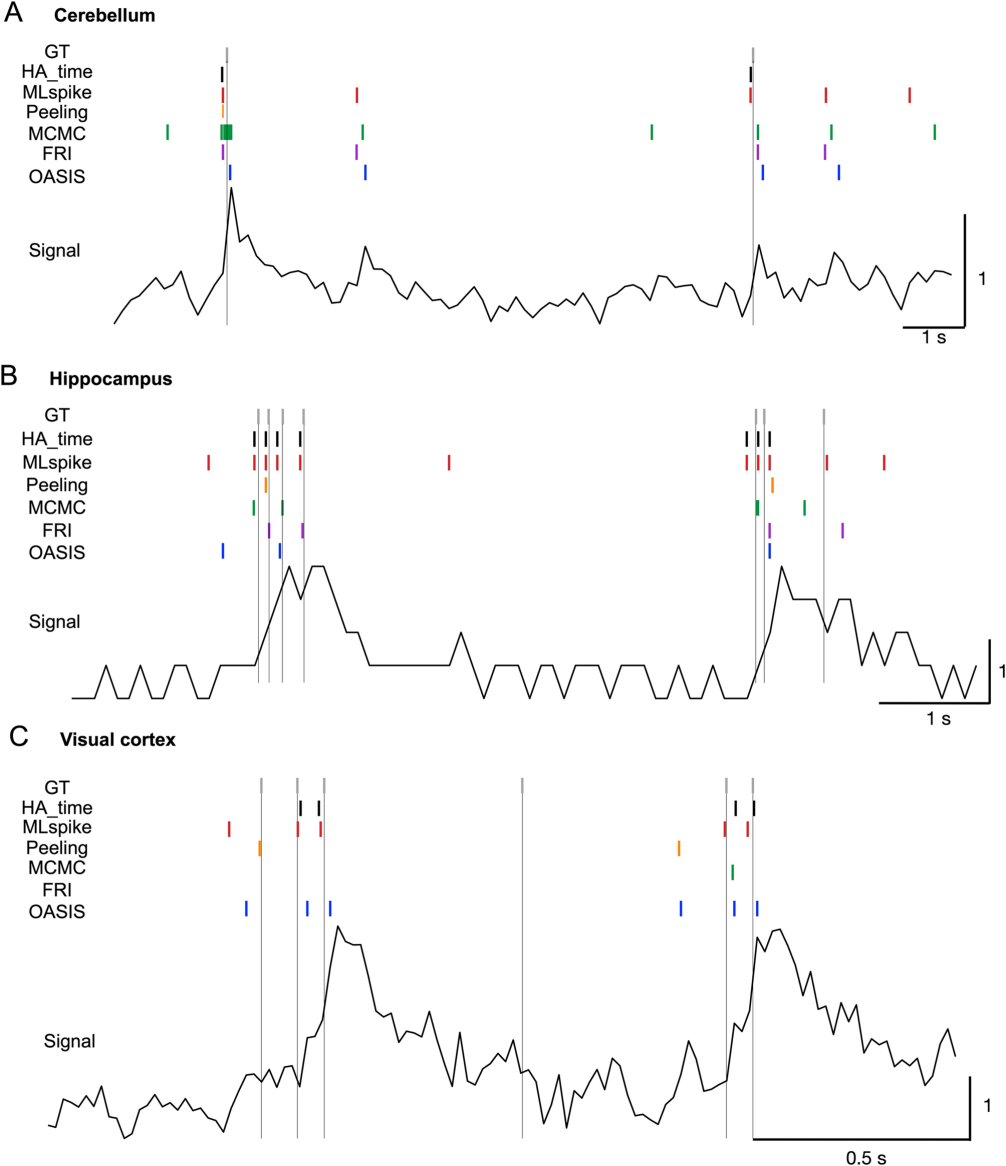
Spike detection by HA_time and benchmark algorithms. Examples of spike detection by HA_time (black bars), MLspike^21^ (red), Peeling^16^ (orange), Monte Carlo Markov Chain (MCMC^22^, green), finite-rate innovation (FRI^17^, purple) and OASIS^15^ (blue) algorithms for the cerebellum (**A**), hippocampus (**B**), and visual cortex (**C**) data. The black traces represent the Ca responses. The thin vertical lines indicate the timing of the ground truth (GT) electrical spikes.

The spikes detected by HA_time (black bars) matched fairly well with the ground truths (gray bars) for all three of the experimental data sets. MLspike (red bars) performed sufficiently for the hippocampus data but rather poorly for the cerebellum and visual cortex data, with many false positives. OASIS (blue bars) performed sufficiently for the visual cortex data but rather poorly for the hippocampus and cerebellum data, with a considerable number of false negatives. The remaining three algorithms (Peeling, orange bars; Monte Carlo Markov Chain [MCMC], green bars; and finite-rate-innovation [FRI], purple bars) performed rather poorly for all three experimental data sets, with many false positives or missing spikes (Fig. 5A–C).

**Figure 6:**
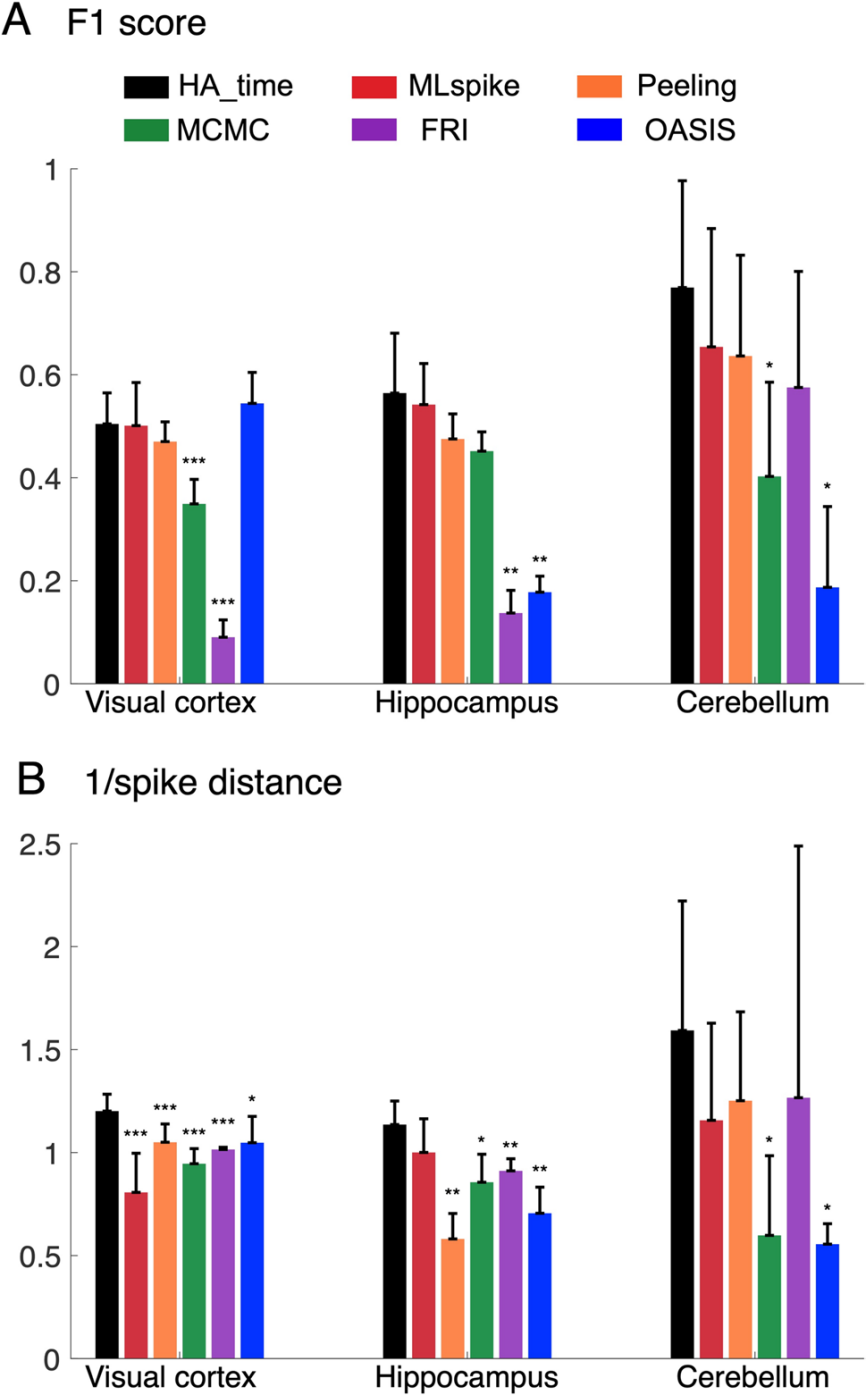
Performance benchmark for experimental data. F1-score (**A**) and inverse of spike distance (**B**) for the HA_time (black columns), MLspike (red), Peeling (orange), MCMC (green), FRI (purple) and OASIS (blue) algorithms. All the algorithms were optimized using the training data (see “Methods). The columns represent means with error bars of + 2SEM. Asterisks indicate the significance level obtained by Wilcoxon signed-rank tests comparing HA_time and the benchmark algorithms. *: p < 0.05; **: p < 0.01; ***: p < 0.001.

We estimated spike detection performance based on the F1 score determined by a receiver operating characteristic (ROC) analysis (see “Methods”). HA_time (0.51 ± 0.06) had one of the best F1 scores for the visual cortex data (0.50 ± 0.08 for MLspike, p = 0.53 for HA_time vs. MLspike; 0.47 ± 0.04 for Peeling, p = 0.1 for HA_time vs Peeling; 0.35 ± 0.05 for MCMC, p = 0.0003 for HA_time vs. MCMC; 0.09 ± 0.03 for FRI, p < 0.0001 for HA_time vs. FRI; 0.54 ± 0.06 for OASIS, p = 0.9 for HA_time vs. OASIS; see Fig. 6A). For the hippocampus data, HA_time (0.56 ± 0.11) performed best, with statistically significant F1 scores compared to those for FRI (0.14 ± 0.04, p = 0.004 for HA_time vs. FRI) and OASIS (0.18 ± 0.03, p = 0.006 for HA_time vs. OASIS), but not in comparison to those for MLspike (0.54 ± 0.08, p = 0.1 for HA_time vs. MLspike), Peeling (0.47 ± 0.05, p = 0.07 for HA_time vs. Peeling), and MCMC (0.45 ± 0.04, p = 0.06 for HA_time vs. MCMC). Similarly, for the cerebellum data, HA_time (0.77 ± 0.2) performed statistically significantly better than OASIS (0.19 ± 0.15, p = 0.03 for HA_time vs. OASIS) and MCMC (0.40 ± 0.18, p = 0.03 for HA_time vs. MCMC), but no statistical significance was found for MLspike (0.65 ± 0.23, p = 0.1 for HA_time vs. MLspike), Peeling (0.64 ± 0.19, p = 0.05 for HA_time vs. Peeling), or FRI (0.57 ± 0.22, p = 0.05 for HA_time vs. FRI). This is probably due to the smaller number of cells (n = 5) in the cerebellum data (Fig. 6A).

The improvement of HA_time in terms of the precision of spike time estimation was also found for the inverse of the spike distance^36^ (see “Methods”). HA_time (1.20 ± 0.08) performed best for the visual cortex data (0.8 ± 0.19 for MLspike, p < 0.0001 for HA_time vs. MLspike; 1.05 ± 0.09 for Peeling, p < 0.0001 for HA_time vs Peeling; 0.94 ± 0.07 for MCMC, p < 0.0001 for HA_time vs. MCMC; 1.01 ± 0.01 for FRI, p < 0.0001 for HA_time vs. FRI; 1.05 ± 0.12 for OASIS, p = 0.02 for HA_time vs. OASIS, Fig. 6B). HA_time (1.14 ± 0.11) also statistically significantly outperformed the benchmark algorithms for the hippocampus data (0.58 ± 0.12 for Peeling, p = 0.004 for HA_time vs Peeling; 0.85 ± 0.13 for MCMC, p = 0.016 for HA_time vs. MCMC; 0.91 ± 0.06 for FRI, p = 0.006 for HA_time vs. FRI; 0.71 ± 0.13 for OASIS, p = 0.004 for HA_time vs. OASIS), except for MLspike (1 ± 0.17, p = 0.1 for HA_time vs. MLspike). Further, for the cerebellum data, HA_time was found to be statistically significantly superior to OASIS (0.55 ± 0.1, p = 0.03 for HA_time vs. OASIS) and MCMC (0.59 ± 0.38, p = 0.03 for HA_time vs. MCMC), but not MLspike (1.15 ± 0.47, p = 0.08 for HA_time vs. MLspike), Peeling (1.25 ± 0.43, p = 0.05 for HA_time vs. Peeling), or FRI (1.26 ± 1.22, p = 0.3 for HA_time vs. FRI).

### Performance evaluation for the simulation data

We further investigated the performance of HA_time in comparison to the benchmark algorithms by simulating the Ca responses sampled in a broader range of conditions than that used for the experiment, including the mean firing frequency of the spike train, the nonlinearity of the Ca responses, the sampling rate of the two-photon recording, the dye dynamics of the Ca responses (time decay constant for the Ca responses) and the SNR (see “Methods”).

We found that the four parameters—mean firing frequency, nonlinearity (see Fig. S2), decay constant, and sampling rate—qualitatively influenced the performance scores of the algorithms, while SNR had only a quantitative influence (see Fig. S3).

**Figure 7:**
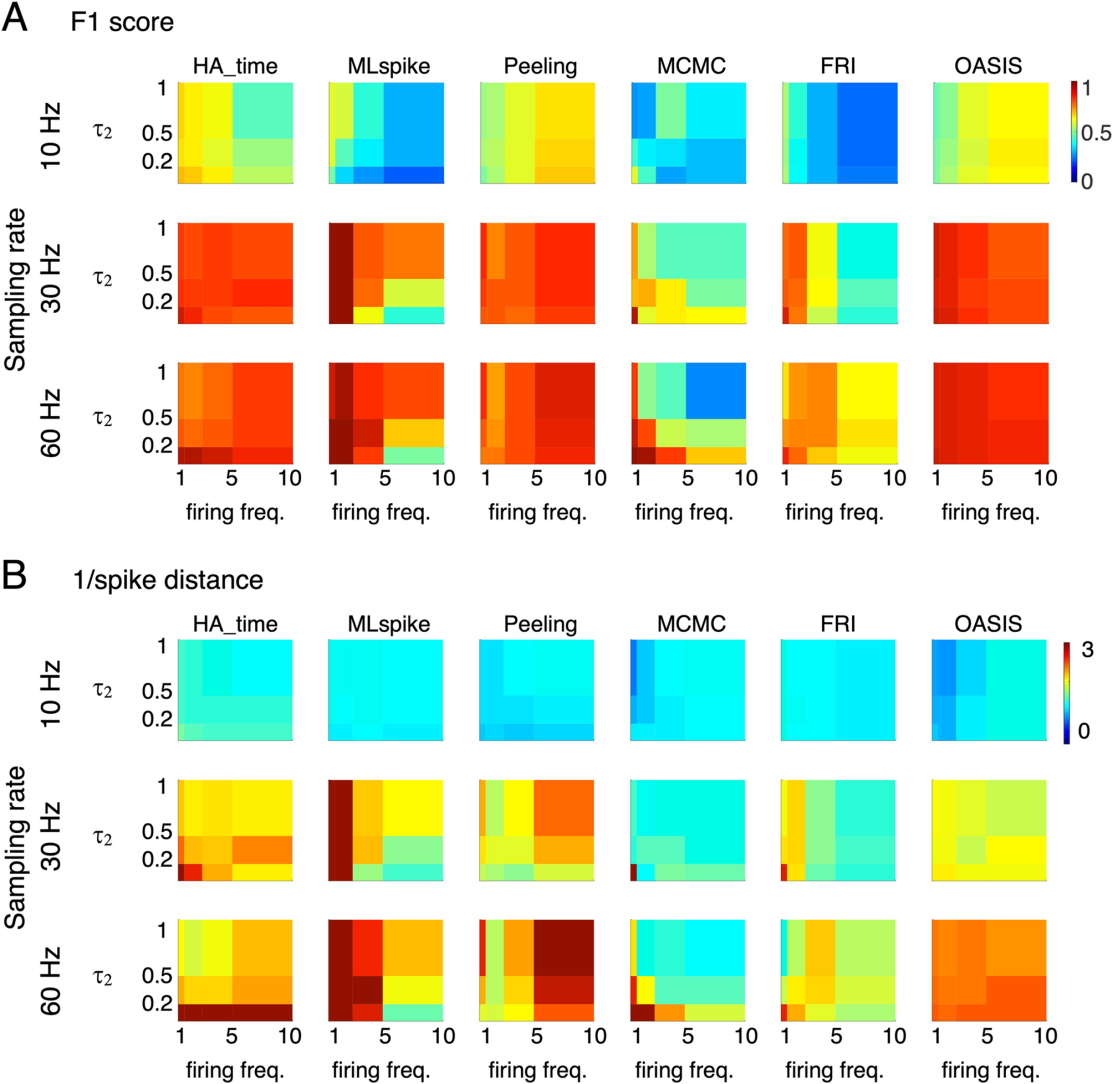
Performance of HA_time and benchmark algorithms for simulation data. 3D pseudo-color maps of the F1 score (**A**) and inverse of spike distance (**B**) as a function of mean firing frequency (abscissa) and decay time constant (τ_2_, ordinate) for the three different sampling rates (10, 30, and 60 Hz). α and SNR were fixed at 1 and 5, respectively.

Figure 7 represents the performance of HA_time as the function of the mean firing frequency and decay time constant (τ_2_) for three different sampling rates (10, 30, and 60 Hz) in 3D pseudo-color maps. HA_time was one of the best algorithms in terms of F1 score across the entire range of parameters (Fig. 7A). MLspike performed best under certain conditions (high sampling rate, 30–60 Hz, low firing frequency, ≤ 2 Hz). Peeling and OASIS performed as well as HA_time in the high sampling rate condition (F1 score > 0.9). MCMC and FRI exhibited sufficient performance only with a high sampling rate, low firing frequency, and fast dye (τ_2_ ≤ 0.5 s).

Spike time precision (1/spike distance) had a performance profile similar to F1 score. Although MLspike performed best with a high sampling rate and low firing frequency and OASIS exhibited sufficient performance with a high sampling rate, the performance of HA_time remained high across the entire range of parameters. Notably, HA_time outperformed all of the benchmark algorithms when a fast dye was used (τ_2_ = 0.2 s, Fig. 7B).

**Figure 8:**
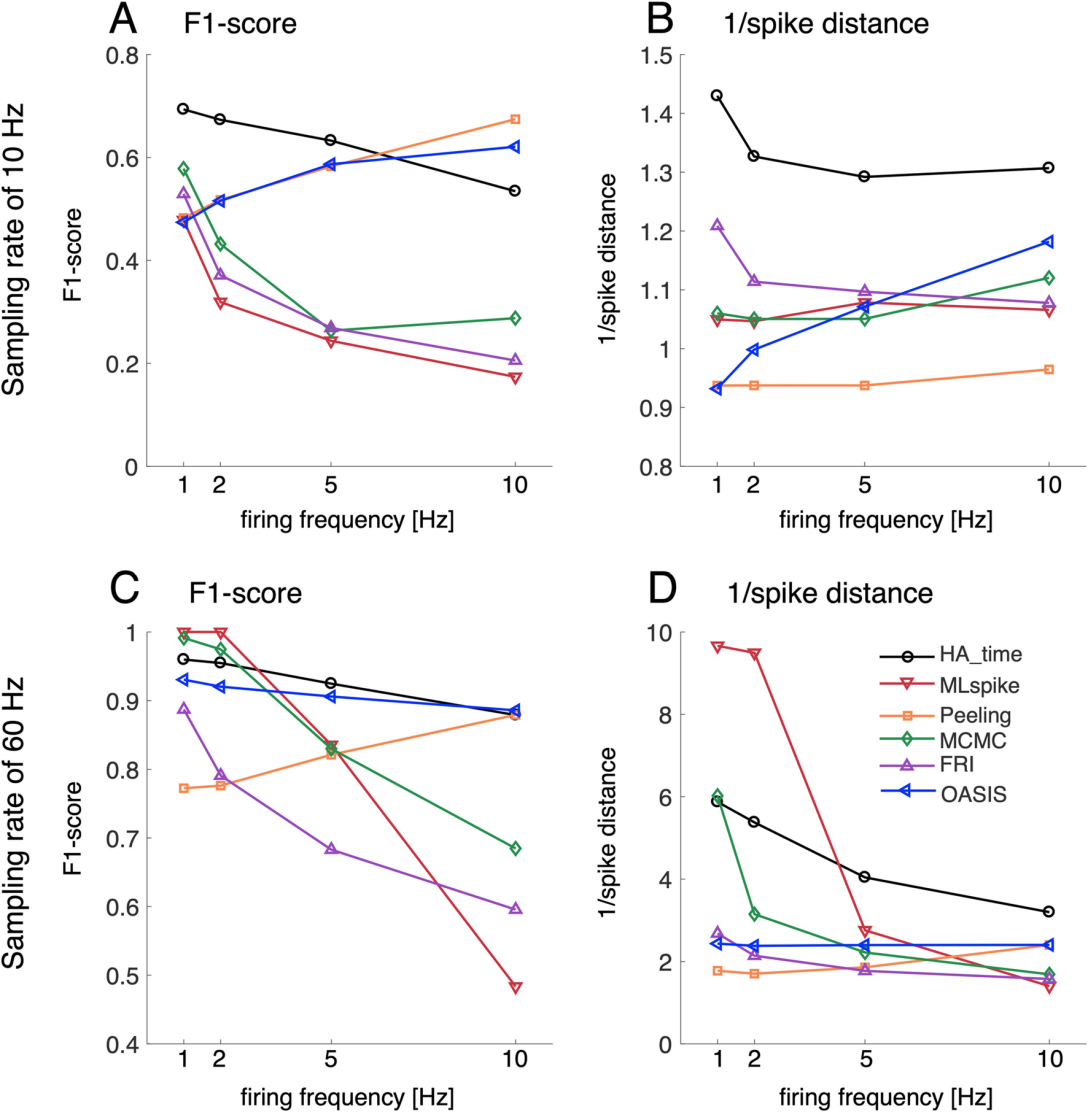
Firing frequency profiles of performance. F1 scores (**A**) and inverse of spike distance values (**B**) for HA_time and the benchmark algorithms as a function of firing frequency at a sampling rate of 10 Hz. C and D are the same measures at 60 Hz. The nonlinearity parameter α, SNR, and τ_2_ were fixed at 1, 5, and 0.2 s, respectively. The color conventions are the same as in Fig. 6.

Figure 8 shows the profiles of the F1 scores and the inverse of the spike distance values for low (10 Hz) and high (60 Hz) sampling rates as a function of the mean firing frequency (τ_2_ = 0.2 s, Fig. 7). HA_time outperformed all the benchmark algorithms under the low sampling rate condition across the entire range of firing frequencies (1–10 Hz, Fig. 8A,B). The advantage of our algorithm over the benchmark algorithms was also demonstrated with a high sampling rate condition across the entire range of spike frequencies. MLspike performed best with a low firing frequency (≤ 2 Hz). However, the performance of MLspike reduced to the level of the other benchmark algorithms as the firing frequency increased (Fig. 8C,D). Peeling and OASIS performed sufficiently in terms of spike detection (i.e., they achieved a relatively high F1 score) but not in terms of spike time precision.

### Unsupervised benchmark for experimental and simulation data

We developed an unsupervised version of HA_time for cases in which simultaneous electrical recordings cannot be obtained. The unsupervised HA_time algorithm is essentially similar to the supervised version, but the Ca spike model parameters are estimated for spike candidates detected by the hyperacuity Bayes method instead of the ground truth spikes (see “Methods”). We generated simulation data for the model parameters and trained the SVM of HA_time with the simulation data. Finally, we selected spikes from the candidates using the trained SVM.

**Figure 9:**
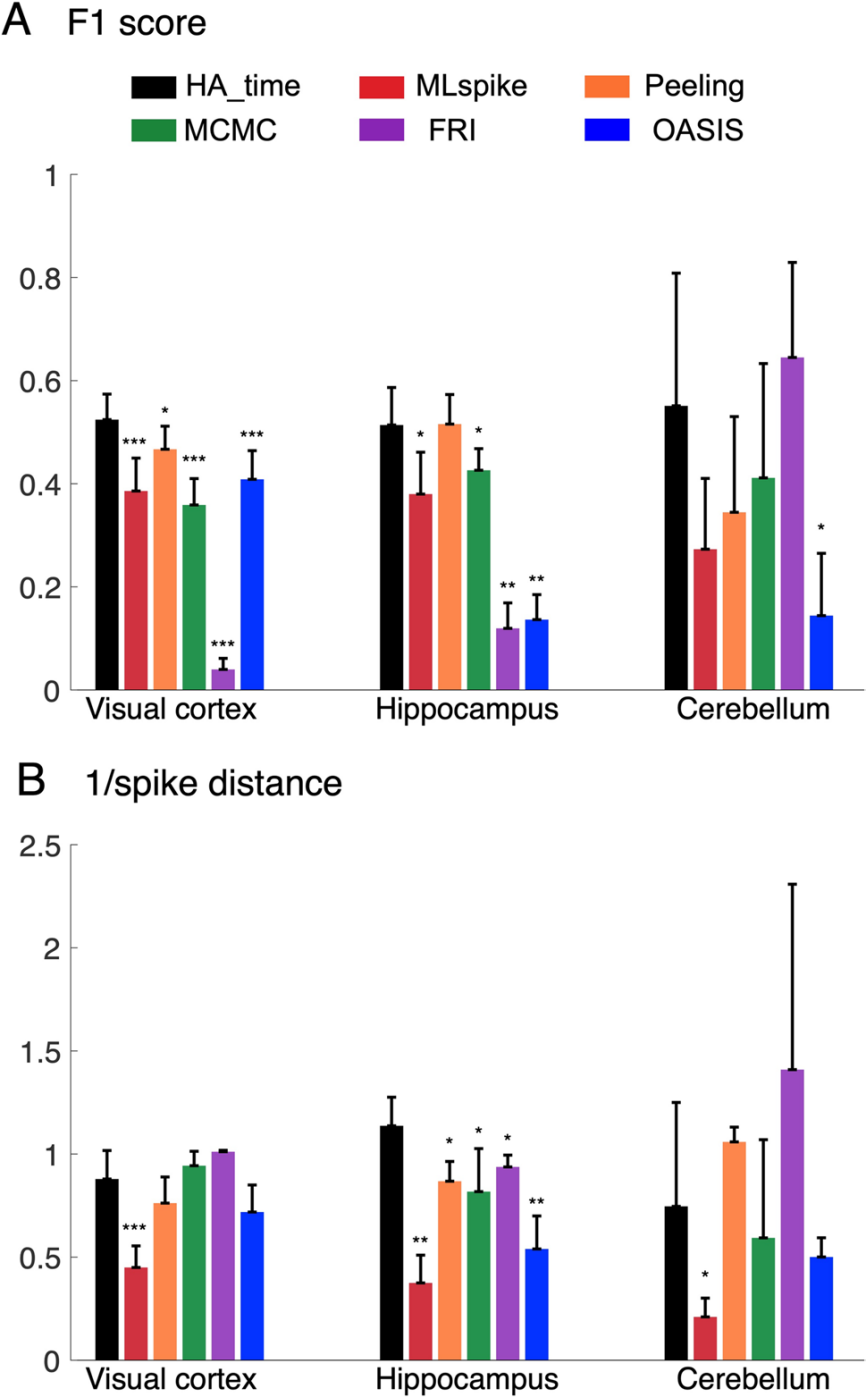
Performance benchmark of unsupervised algorithms for experimental data. F1 score (**A**) and inverse of spike distance (**B**) for unsupervised HA_time and the unsupervised benchmark algorithms. The conventions are the same as in Fig. 6.

Unsupervised HA_time maintained sufficient F1 scores for the visual cortex data, which included a relatively large number of cells. HA_time was the best algorithm in terms of F1 scores for the visual cortex data (0.5 ± 0.05 for HA_time; 0.38 ± 0.06 for MLspike, p = 0.0002 for HA_time vs. MLspike; 0.46 ± 0.04 for Peeling, p = 0.01 for HA_time vs Peeling; 0.35 ± 0.05 for MCMC, p = 0.0006 for HA_time vs. MCMC; 0.04 ± 0.02 for FRI, p < 0.0001 for HA_time vs. FRI; 0.4 ± 0.05 for OASIS, p = 0.0004 for HA_time vs. OASIS, Fig. 9A). It was also superior for the hippocampus data, except in comparison to Peeling (0.51 ± 0.07 for HA_time; 0.38 ± 0.08 for MLspike, p = 0.01 for HA_time vs. MLspike; 0.51 ± 0.06 for Peeling, p = 0.6 for HA_time vs Peeling; 0.42 ± 0.04 for MCMC, p = 0.03 for HA_time vs. MCMC; 0.12 ± 0.05 for FRI, p = 0.004 for HA_time vs. FRI; 0.14 ± 0.05 for OASIS, p = 0.004 for HA_time vs. OASIS). The F1 score of HA_time (0.55 ± 0.25) was second highest for the cerebellum data, but without statistical significance (0.27 ± 0.13 for MLspike, p = 0.05 for HA_time vs. MLspike; 0.34 ± 0.18 for Peeling, p = 0.17 for HA_time vs Peeling; 0.41 ± 0.22 for MCMC, p = 0.08 for HA_time vs. MCMC; 0.64 ± 0.18 for FRI, p = 0.94 for HA_time vs. FRI; 0.14 ± 0.12 for OASIS, p = 0.02 for HA_time vs. OASIS). Regarding spike time precision (1/spike distance), we found unsupervised HA_time had less marked advantages over the unsupervised benchmark algorithms, with weaker statistical significance than for F1 score (see Fig. 9B).

We also conducted a similar analysis for simulation data (τ_2_ = 0.5 s, sampling rate, 10 Hz). We confirmed the advantages of unsupervised HA_time over the unsupervised benchmark algorithms for spike detection and spike time precision (Fig. S4). The results were comparable to those obtained for the experimental data.

## Discussion

### Hyperacuity spike-time estimation algorithm combining the generative model and supervised learning approaches

HA_time is an example of the so-called super-resolution techniques, which have proven successful in many fields of signal processing^37,38^. Such techniques use a forward model for signal distortion due to sensing and sampling and recover the lost information by estimation of the model parameters maximizing the likelihood of the estimates. We used a double exponential function as the forward model to represent the dynamics of Ca dyes, a method that has been widely used in neuroscience^3–5^, and Bayesian inference, which is a common way to maximize the likelihood of estimation of model parameters. The accuracy of spike detection was further enhanced by an SVM trained with the ground truth. We found that HA_time improved the precision of spike time estimation fourfold (range, two–sevenfold; 2SEM, 0.1–0.25) compared to the sampling rate, across a wide range of sampling rates and other parameters. The recent progress in two-photon imaging with fast and linear Ca dyes^39^ can use the advantages of hyperacuity algorithms to relax the trade-off between the sampling rate and the number of sampled neurons.

HA_time aimed to resolve two challenging issues – reliable spike detection and enhanced spike time precision—in the presence of the nonlinearity, slow dynamics, and low SNR of Ca imaging data. The difficulty of achieving this goal arose from the spike dynamics of the target neurons and/or kinematics of the Ca responses, which may vary dramatically across brain regions. The slow Ca kinematics and low SNR may limit the temporal precision of the information conveyed by the Ca imaging data. HA_time overcame this difficulty by combining a generative model with a supervised classifier. It estimated the Ca spike model using Bayesian inference assuming that spikes have a constant size and shape, compensating for the nonlinearity of the Ca responses by nonlinearity analysis, and detecting the spikes from the compensated Ca imaging data using the ground truths as supervised information. Hyperacuity estimation of spike time was achieved by recalibrating the spike time using the hyperacuity vernier scale to minimize the residual errors in the Ca response model prediction. Our combined approach may improve the performance of HA_time in two ways: the Ca response model enhanced spike detection and spike time estimation with higher temporal precision than the sampling resolution, while supervised learning compensated for fluctuations in the Ca imaging data caused by noise and sampling jitters, which are not considered by the generative model.

We also developed a generative model algorithm, hyperacuity Bayes (see “Methods" and Supplementary Information), which is a partial algorithm of HA_time. In this algorithm, the estimation of the generative model by Bayesian inference based on ground truth information was very robust. However, we found that the combination approach (i.e. HA_time) was superior for estimating spike timing compared to the hyperacuity Bayes algorithm, as evidenced by the significantly higher F1 score and inverse of the spike distance for the hippocampus and visual cortex data (see Fig. S5).

Notably, HA_time exhibited reasonable performance even for the experimental data with no ground truths (i.e., simultaneous electrical recordings). Unsupervised HA_time estimated the model parameters and detected spike candidates for experimental data using hyperacuity Bayes. The SVM was trained with the simulation data generated for the estimated model parameters, and then used to select spikes from the set of candidates. Unsupervised HA_time maintained performance comparable to the supervised version for all the experimental data sets (see Figs. 6 and 9). This fact indicates that the training of the SVM with simulation data extended to spike detection for experimental data. Thus, HA_time has good generalizability and is practical for precise spike timing estimation from two-photon imaging data with no ground truth information.

We realized a potential risk where the nonlinearity of Ca responses reported for strong burst activity by two-photon imaging studies of the cortical activity^35,39^ may impair the performance of HA_time. However, we found that spikes of such strong burst activity (number of spikes > 6 and spike frequency > 10 Hz) only occupied a small fraction of all of the visual cortical spike samples (less than 10%). Therefore, this effect would be rather small. This view is consistent with the finding that HA_time performed best for the visual cortical data across all of the hyperacuity algorithms.

### Performance evaluation in comparison with the benchmark algorithms

We computed the three metrics to evaluate the algorithm’s performance. The F1 score of the ROC analysis increased for hits and decreased for both false positives and negatives in spike detection. The inverse of spike distance (when the weighted cost of shifting spikes is proportional to the time window of spike detection) measured the temporal precision of detected spikes. The CosMIC metric assessed the similarity of the estimations with the ground truth spike trains by convoluting the two spike trains with a smooth kernel^40^ (see “Methods”). We found that CosMIC exhibited performance profiles quite similar to those for the F1 score, with a strong correlation (r = 0.96, see Fig. S6). Thus, we used the F1 score and inverse of spike distance as the metrics for evaluating spike detection and spike time precision, respectively.

We benchmarked HA_time and the other five algorithms for the three biological data sets with different data conditions. In terms of the F1 score, we found that HA_time performed best with sufficient statistical significance in the visual cortex and hippocampus data, which included a relatively large number of cells. However, for the cerebellum data, in which the number of cells was rather small (n = 5 cells), HA_time statistically outperformed only the OASIS and MCMC algorithms. HA_time also outperformed all benchmark algorithms in terms of the precision of spike time estimation, which was estimated as the inverse of the spike distance, with statistical significance for the visual cortex and hippocampus data. Similarly, HA_time outperformed only MCMC and OASIS for the cerebellum data.

We conducted a systematic study of the algorithm's performance for simulation data that covered a broader range of parameters than the experimental conditions, including the mean neuronal firing frequency, nonlinearity, decay time of the Ca dyes, SNR and the sampling rate. The F1 scores and the inverse of the spike distance values, which were studied as the functions of those parameters, indicated that mean firing frequency, nonlinearity, decay time constant (τ_2_) and sampling rate were the parameters with the greatest influences on performance. SNR was the least important parameter, influencing only size and not significantly changing the shape of the performance functions. The F1 score and the inverse of spike distance functions highlighted the superiority of HA_time over the other algorithms, where it obtained high scores with spike detection and high spike time precision across the entire range of parameters. However, in the condition with a high sampling rate and low firing rate, MLspike slightly outperformed HA_time. The performance of the other benchmark algorithms remained sufficient only in the condition of weak nonlinearity, high sampling rate, and low firing frequency. Conversely, HA_time maintained high performance in the conditions with strong nonlinearity and/or high firing frequency, which are frequently encountered in the cortical cells of behaving animals. It should be emphasized that HA_time exhibited practically meaningful hyperacuity performance for the fast dye with a small decay constant (τ_2_ = 0.2 s) across a wide range of nonlinearity and firing frequency conditions. Thus, it may be useful for two-photon recording studies of cortical neurons using fast dye dynamics. The simulation analysis of the performance of HA_time and the benchmark algorithms across a wide range of experimental conditions for two-photon recordings may provide useful information for selection of the best algorithm for certain experimental conditions.

## Methods

### Hyperacuity support vector machine (HA_time)

HA_time detects spikes contained in the Ca responses of two-photon recordings in three steps: (1) estimation of the Ca response model using the expectation–maximization (EM) algorithm assuming a constant shape and size of the spike model, (2) detection of spikes in the Ca responses by an SVM assisted by the matching information of the Ca response and Ca spike model, and (3) estimation of spike time for the detected spikes to minimize the errors between the Ca response model prediction and the Ca imaging data using the vernier scale, which is 10 times finer than the sampling interval of two-photon recordings.

#### Ca spike model estimation by Bayesian inference

We estimated the parameters of the Ca response model assuming that all of the Ca responses in the two-photon recording originated from a unique Ca spike model *g(t, T,* τ*)* and will vary due to the noise and sampling jitters (SJs)^19^.

$$\begin{aligned} g(t,T,\tau ) & = \left( {1 - \exp \left[ {\frac{T - t}{{\tau_{1} }}} \right]} \right)\exp \left[ {\frac{T - t}{{\tau_{2} }}} \right],\,\,t \ge T \\ g(t,T,\tau ) & = 0,\,\,t < T \\ \end{aligned}$$

where t, T, τ = (τ_1_, τ_2_) are the time, the spike onset, and the rise and decay time constants, respectively.

We estimated a Ca response model whose parameters are the model amplitude (a), baseline (b_0_) and noise (σ) using the EM algorithm, while the time constants of spike response (τ) were estimated by an iterative alternate coordinate one-dimensional grid search (see Supplementary Information).

#### Spike detection by SVM

We conducted spike detection and spike time estimation by an SVM supplemented with information from the Ca response model. We estimated the coincidence scores, determined as the convolution (dy/dt * dg/dt) of the first-order derivative (*dy/dt*) of the Ca signals of a two-photon recording with the first-order derivative (*dg/dt*) of the Ca response model (*g),* estimated by Bayesian inference for the training data. For the spike candidates, we sampled data segments that exceeded the threshold. The threshold and the length of data segments (the number of data points before and after the point exceeding the threshold) were optimized according to the F1 score for the training data (see the discussion of cross-validation in Statistical Analysis). The SVM was trained to classify spike candidates into spikes or non-spikes by feeding in the spike candidates and coincidence scores as the primary and attribute inputs, respectively, and the electrical spikes (ground truth) as the teaching signals.

#### Hyperacuity spike time estimation

The trained SVM was used for detection of spikes in the test data. The pseudo-spike times (PTs) were tentatively determined for the detected spikes as those for which the coincidence score exceeded the threshold. We assumed that the PTs may vary due to the SJs (difference between pseudo- and true spike times), and thus we estimated the SJs to minimize the prediction errors between the Ca response and the Ca response model by systematically changing the SJ according to a vernier scale 10 times finer than the sampling interval. The true spike time (TT) was calculated as the sum of PT and SJ (see the inset of Fig. 1C). For spike time estimation in cases where the preceding spikes overlapped with the succeeding ones, we subtracted the trace of the preceding spike from the trace of the succeeding one.

#### Unsupervised HA_time

We developed an unsupervised version of HA_time for cases in which the ground truth signals are unavailable. First, we estimated the Ca response model parameters – including spike amplitude, time constants (τ_1_ and τ_2_), noise level and mean firing rate – from the Ca imaging data using the hyperacuity Bayesian algorithm (see Supplementary Information). Then, we generated simulation data for the model parameters and trained the SVM with the simulation data. Next, we detected the spike candidates in a similar way as the supervised cases. The SVM was used to select spikes from the set of candidates, and finally, spike time was estimated using the same jittering processing as was used for the supervised algorithm.

### Other benchmark algorithms

We evaluated the performance of four algorithms that aim for hyperacuity: MLspike^21^, Peeling^16^, the finite-rate innovation method (FRI)^17^, and the Monte Carlo Markov chain (MCMC)^22^. In addition, we evaluated a recently developed deconvolution algorithm that does not aim at hyperacuity (OASIS)^15^. These evaluations were performed for supervised and unsupervised cases in which algorithm parameters were optimized and not optimized, respectively, using ground truth signals.

In the supervised cases, MLspike used the ground-truth signals (i.e., electrical spikes) for optimization of the parameter set, including the spike amplitude, time constants of Ca dye, nonlinearity parameters, Hill coefficient, and spike time delay. As neither the Peeling nor the FRI method includes a routine to optimize the parameters, we supplemented them with the parameter settings used for the Ca response model of HA_time. MCMC was used to estimate the parameters directly from the Ca imaging data, and we supplemented our estimations as the parameters’ initial values. Unlike the other hyperacuity algorithms, which directly output the spike times, OASIS predicted the deconvoluted trace correlated with the underlying spike train. We used a threshold to determine the spike time based on this deconvoluted trace. The threshold and the sparsity parameter of OASIS were optimized in the ranges of 0–5 SD and 1–10, respectively, in order to maximize the F1 score of the training data.

In unsupervised cases where no ground truth information was given, the parameters of the Peeling and FRI algorithms were set to the values reported in the papers in which they were introduced^16,17^. The other three algorithms – MCMC, MLspike and OASIS – used their own routines to estimate the parameters. The threshold for determining the spike time by OASIS was fixed at 2 SD.

### Experimental data sets

We collected simultaneous electrical and two-photon recordings of the Ca signals in three cortical areas (cerebellum, hippocampus and visual cortex) using five different Ca dyes as described below (see Table S1 for details). The experiments for the hippocampus and cerebellum data sets were approved by the Animal Experiment Committees of the University of Tokyo and University of Yamanashi. All experimental protocols were carried out in accordance with the Fundamental Guidelines for Proper Conduct of Animal Experiment and Related Activities in Academic Research Institutions (Ministry of Education, Culture, Sports, Science and Technology, Japan). The approval for the visual cortex experiments were described in refs 35,41.

#### Recording of cerebellar Purkinje cell complex spikes

We collected experimental data for the complex spikes of five cerebellar Purkinje cells from the work of^33^, where simultaneous two-photon Ca imaging (sampling rate, 7.8 Hz) using multicell bolus loading of Cal-520 dye and extracellular recording (sampling rate, 20 kHz) was performed on adult mice.

#### Recording of hippocampal CA3 neurons

We collected the simultaneous cell-attached recording (sampling rate, 20 kHz) and one-photon images (10 Hz) of Ca responses from nine CA3 pyramidal neurons in organotypic cultured slices of rats stained with OGB-1AM dye^34^. The Ca signals were normalized by the peak of the Ca spike model estimated for individual cells.

#### Recording of the primary visual cortex

We collected the three data sets recorded from the mouse visual cortex using different Ca indicators (GCaMP6f, GCaMP6s, GCaMP5k). All included simultaneous loose-seal cell-attached patch recordings (sampling rate, 20 kHz) and two-photon recordings of Ca responses (sampling rate, 50–60 Hz, see 1–3 in Table S1)^35,41^.

### Simulation data

We conducted a simulation of the Ca responses for the three experimental data sets (i.e., cerebellum, hippocampus, and primary visual cortex). Spike events were generated according to a Poisson distribution, with the mean firing rate varying from 1–10 Hz. The Ca responses were simulated by convolving the double exponentials with time constants for rise and decay with the spike events. The rise time constant τ_1_ was fixed at 0.01 s, while the decay time constant τ_2_ was varied from 0.2–1 s, corresponding to those of the OGB-1AM, Cal-520, and GCaMP6f dyes. We introduced the parameter α to reproduce the nonlinearity found in the Ca responses *f(t)* in the three cortices as follows:

$$f\left( t \right) \, = \, x\left( t \right)^{\alpha } ,\,\, for\, \, x\left( t \right) > 1$$

$$f\left( t \right) \, = \, x\left( t \right), \, otherwise,$$

where *x(t)* = g(t) * s(t) is the linear response of the Ca spike model given the spike train *s(t)*. The parameter α for saturation (α < 1) and superlinearity (α > 1) varied in the range of 0.2–3, based on the values found in the three experimental data sets. Finally, Gaussian noise was added to reproduce the SNR (3, 5, 10) of the experimental data. For each set of simulation parameters, 500 spike signals were generated in a total of 10 cells, and those from five cells were used as the training and test data sets.

### Performance analysis

To evaluate spike detection performance, a correct hit case was defined as one in which the time difference between an estimated spike and a true one was smaller than a time window of half the sampling interval. The opposite was true for missing cases. A false positive case was defined as one in which the time difference between the true spike and the estimated one was greater than the time window. For data sets with a high sampling rate (30–60 Hz), the time window was relaxed to 50 ms.

The receiver operating characteristic (ROC) analysis was conducted as follows:

$$\begin{aligned} {\text{Sensitivity}} & = {\text{Hit}}/\left( {{\text{Hit}} + {\text{misses}}} \right) \\ {\text{Precision}} & = {\text{Hit}}/\left( {{\text{Hit}} + {\text{False}}\,{\text{positive}}} \right) \\ {\text{F1 score}} & = {2} \times \left( {{\text{Sensitivity}} \times {\text{Precision}}} \right)/\left( {{\text{Sensitivity}} + {\text{Precision}}} \right) \\ \end{aligned}$$

We estimated the temporal precision of spike time estimation as the inverse of the spike distance, defined as the minimal cost for reconstructing the true spike train from the estimated one. We allowed one each for deletion or insertion of the spike event and the weighted cost of the shift in spike time^36^. To emphasize the precision of spike time estimation, the weighted cost of shifting spikes was equal to the inverse of the time window for accepting hit cases. The spike distance was further normalized by the number of ground truth spikes.

We computed the CosMIC metric to determine spike time precision^40^. The ground truth and estimated membership functions – *y(t)* and *y*_*est*_*(t)*, respectively – were computed by convoluting the ground truth and estimated spike train – *s(t)* and *s*_*est*_*(t)*, respectively – with the triangle kernel *p(t)*:

$$\begin{array}{*{20}c} {y\left( t \right) = s\left( t \right) *p\left( t \right)} & {y_{est} \left( t \right) = s_{est} \left( t \right) * p\left( t \right)} \\ {p\left( t \right) = \frac{e - \left| t \right|}{e}, \left| t \right| < e} & {p\left( t \right) = 0, otherwise} \\ \end{array} ,$$

where *e* is the width of the triangle kernel *p(t)*, and is equal to the time window of the ROC analysis. The CosMIC metric was then computed as follows:

$$CosMIC =2\frac{|| min(y, {y}_{est})||}{(||y|| + ||{y}_{est}||)}$$

We also evaluated the improvement of spike time precision as the hyperacuity index, estimated as the ratio of the sampling interval to the mean spike time errors (i.e., difference between the estimated and ground truth spike times) for hit cases.

### Statistical analysis

Performance analyses of all algorithms applied to the experimental data were conducted with leave-one-out cross-validation, and the data from one cell and remaining cells were used for testing and training, respectively.

All of the performance scores were estimated as the mean ± 2SEM. To assess statistical significance, we compared the performance of HA_time to that of the benchmark algorithms using a one-sided Wilcoxon signed-rank test, and we reported the significance level (p).

### Hyperacuity Bayesian algorithm

We also developed a hyperacuity Bayesian algorithm by essentially creating an algorithm similar to that for HA_time for cases in which no ground truth signals are available. This algorithm maximizes the likelihood for the Ca signals recorded by two-photon recordings (see Supplemental Information).

## Supplementary information

Supplementary information

## Data Availability

The MATLAB implementation of our algorithm can be found online (https://github.com/hoang-atr/HA_time). The hippocampus and cerebellum data sets used in this work are available from the authors upon request.
