# Supplementary material for: Improved hyperacuity estimation of spike timing from calcium imaging
Source: Sci Rep. 2020 Oct 20;10:17844. doi: 10.1038/s41598-020-74672-y (PMC7576127; doi:10.1038/s41598-020-74672-y)
Supplement: Supplementary file 1 — Supplementary information [file 41598_2020_74672_MOESM1_ESM.docx]

**Improved hyperacuity estimation of spike timing from calcium imaging**

Huu Hoang^1*^, Masa-aki Sato^1*^, Shigeru Shinomoto^1,2^, Shinichiro Tsutsumi^3^, Miki Hashizume^4^, Tomoe Ishikawa^5^, Masanobu Kano^3^, Yuji Ikegaya^5^, Kazuo Kitamura^6^, Mitsuo Kawato^1^, Keisuke Toyama^1#^

^1^ATR Brain Information Communication Research Laboratory Group, Advanced Telecommunications Research Institute International, Kyoto, Japan

^2^Department of Physics, Kyoto University, Kyoto, Japan

^3^Department of Neurophysiology, Graduate School of Medicine, The University of Tokyo, Tokyo, Japan

^4^Department of Biochemistry, Faculty of Medicine, Saitama Medical University

^5^Graduate School of Pharmaceutical Sciences, The University of Tokyo, Tokyo, Japan

^6^Department of Neurophysiology, Faculty of Medicine, University of Yamanashi, Yamanashi, Japan

* Equally contributing first authors

# Correspondence: Keisuke Toyama, toyama@atr.jp

# Supplementary Information

## Hyperacuity Bayesian algorithm

We developed a hyperacuity Bayesian (HB) algorithm for spike detection and spike time estimation that maximizes the estimated likelihood for the cases where ground truth signals are not available. Both supervised and unsupervised versions of the HB algorithm were developed. The supervised version reproduces procedures similar to those for HA_time, such as estimation of the Ca response model, spike detection, and spike time estimation using the model information. Probabilistic models and the EM algorithm are described in the first two sections, and detailed procedures for the two versions of the HB algorithm are described in the next two sections.

### Data structure and probabilistic model

Let us suppose that *K* data segments were sampled from data by thresholding, while leaving the rest of the data (*y_rest_*):


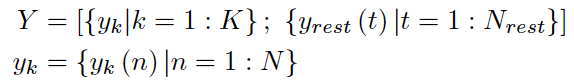


where *y_k_(n)* is the sampled data at the sampling time *t_k,n_= (n−1)dt_0_+t_k,1_* of the *k*-th window, and *dt_0_* is the sampling step of the observed data with the sampling frequency, defined as *f_0_ = 1/dt_0_*.

A probabilistic model for spike states is as follows:


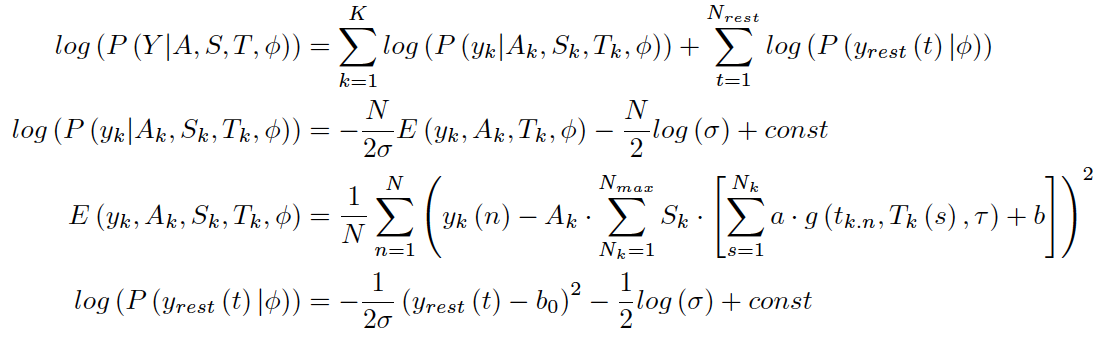


where *A_k_* is a spike indicator variable that represents the presence or absence of spikes in the *k*-th window and which takes a binary value (0 or 1). *S_k_* represents a spike state in the *k*-th window and takes a binary vector value (Potts spin variable).


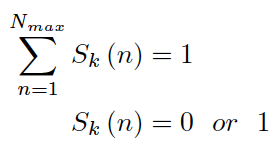


*S_k_(N_k_) = 1* means that there are *N_k_* spikes in the *k*-th window. The maximum number of spikes in a window is assumed to be *N_max_*. *T_k_* refers to a set of spike times in the *k*-th window, and *T_k_(s)* is the onset time of the *s*-th spike. In addition, *a* represents the amplitude of the spike response function, while *b* and *b_0_* represent the bias in spike and no spike regions, respectively, and *σ* is the variance of the Gaussian noise. A set of global parameters, which are assumed to be common for all spikes and the rest of the data, is denoted by
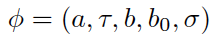
.

We assume the hierarchical noninformative priors as follows:


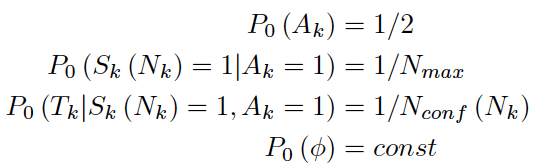


where *N_conf_(N_k_)* represents the number of configurations of *T_k_* for the *N_k_* spike case.

### Expectation-maximization (EM) algorithm

In the E-step, the posterior probability of the spike state for the current estimate of the model parameters phi for combination of the log-likelihood and the noninformative prior, the joint probability for a spike state in the k-th window, are given by the following:


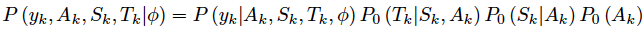
,

and marginal probability is given by


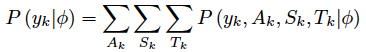
.

The posterior probability for a spike state is then calculated as follows:


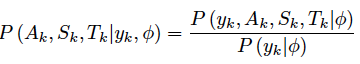
.

In the M-step, the model parameters were updated to the new value
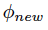
 by maximizing the Q-function, defined as follows:


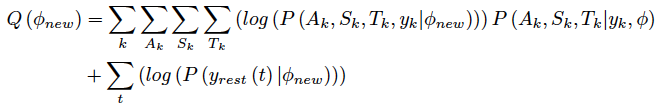


by solving the maximum condition


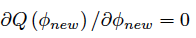
.

### Spike estimation for the supervised version of HB

We extracted continuous regions in which the signals exceed the threshold. The threshold was optimized to maximize the true positive cases and minimize the false positive cases referring to the ground truth given by the electrical spikes. Each region was then segmented into fixed-length data segments comprised of eight points. We divided two overlapping data segments, whose onset intervals were less than the length of the data segment (8 points), into three divided nonoverlapping segments. For example, two segments of points 1–10 and 5–14 were divided into three non-overlapping segments of points 1–4, 5–10, and 11–14.

Next, we utilized the training data to estimate the model parameters and the classifier for posterior probability. The spike model parameters, including the spike amplitude (*a*), biases (*b, b_0_*), and noise variance (*σ*), were estimated by the EM algorithm (see prior section), while the time constants of the spike response (τ) were estimated by an iterative alternate coordinate one-dimensional grid search because the log-likelihood with respect to τ is highly nonlinear. The maximum number of spikes contained in single data segments was estimated for the 95th percentile value of the spike number histogram of the training data segments. The posterior probability of the spike state for the training data was estimated for each data segment assuming that they are independent of each other. For overlapping segments, posterior probabilities were integrated with the overlapping segments by Bayesian inference. We used a multinomial classifier to predict the number of spikes based on the posterior probability.

Finally, we estimated the spike number and spike onset time for the test data. The data segments were sampled from the test data by thresholding. The threshold was optimized for the training data, and the posterior probability of the spike state for each segment was calculated in the same way as for the training data. The number of spikes was estimated based on the posterior probability for the number of spikes using the multinomial classifier trained for the training data, and spike onset times were estimated with the hyperacuity time step by maximizing the log-likelihood of the estimated number of spikes. The contributions of the preceding spikes were subtracted from the data signal, as was done for HA_time.

### Spike estimation by the unsupervised version of HB

The unsupervised version of HB was used to perform spike estimation in essentially the same way as that for the supervised version, except for the algorithm used to estimate the spike state. We conducted Bayesian inference assuming that the initial spike state for each data segment contains only one spike whose waveform represents the spike response. We optimized the threshold and τ = (τ_1_, τ_2_) to maximize the log-likelihood and estimated the number of spikes for each data segment that gave the maximum posterior probability for the given number of spikes.

**Supplementary Figures**


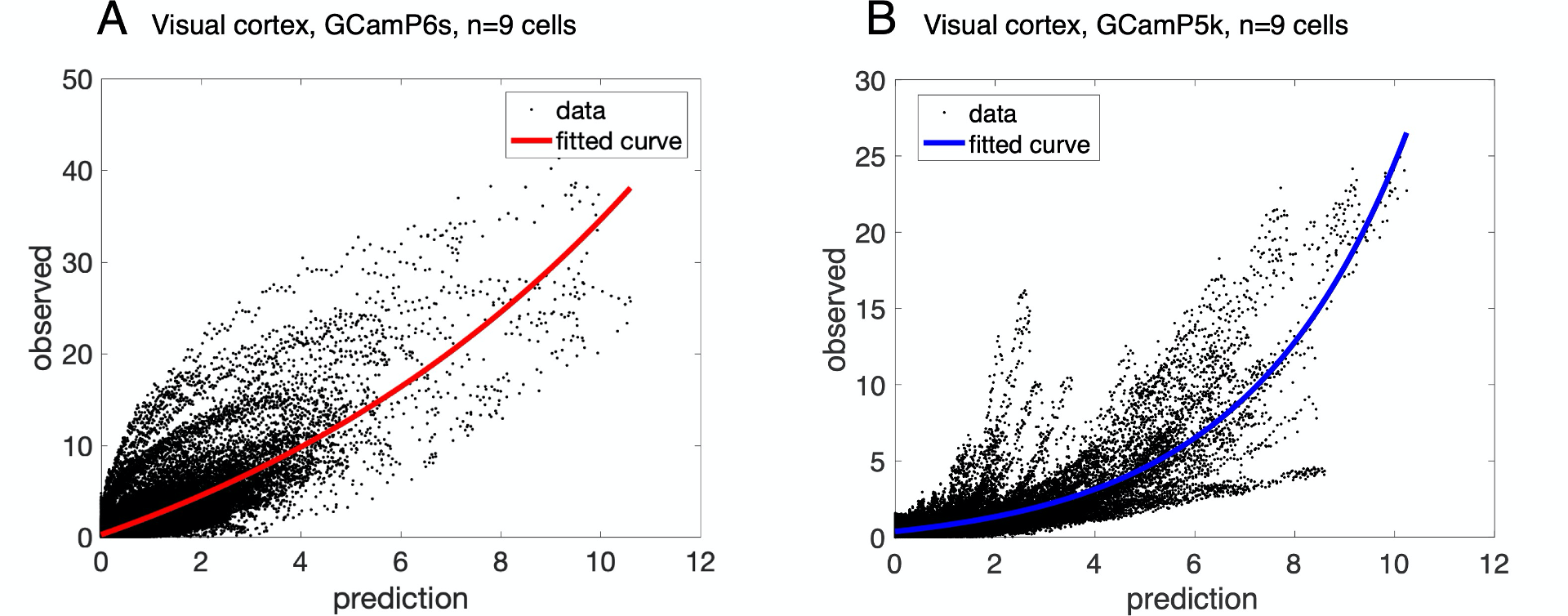


**Supplementary Figure S1: Nonlinearity analysis of the visual cortex data.** Scatter diagrams of the Ca imaging data in the two visual cortex data sets, with **(A)** GCaMP6s and **(B)** GCaMP5k as a function of the linear prediction of the Ca response model for spike trains.


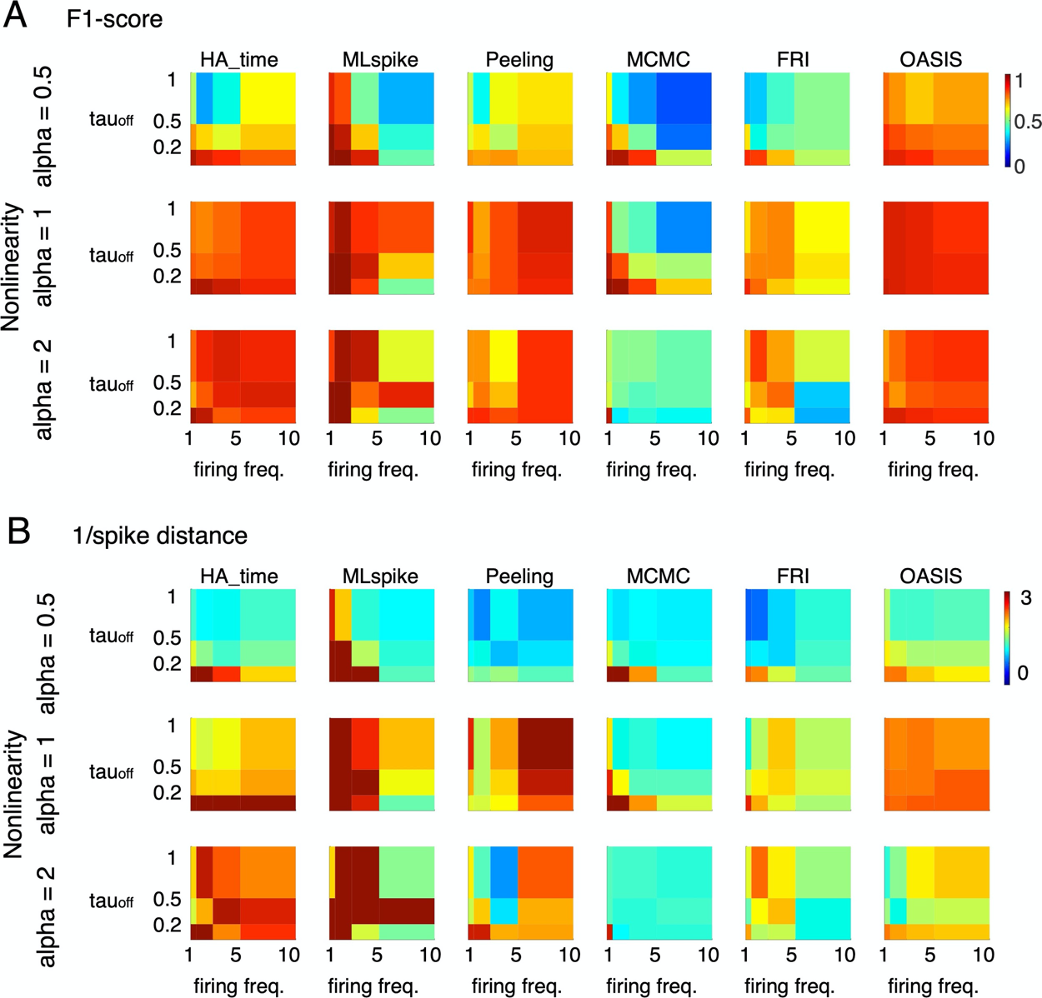


**Supplementary Figure S2:** Performance of HA_time and benchmark algorithms for simulation data with variation in the nonlinearity of the Ca response model. **(A)** F1 score and **(B)** inverse of spike distance for three different nonlinearity parameters (α = 0.5, 1, 2) of the Ca response model (τ_2_ = 0.2 s). The ordinate, abscissa, and calibration scales of the pseudo-color maps are the same as in Fig. 7.


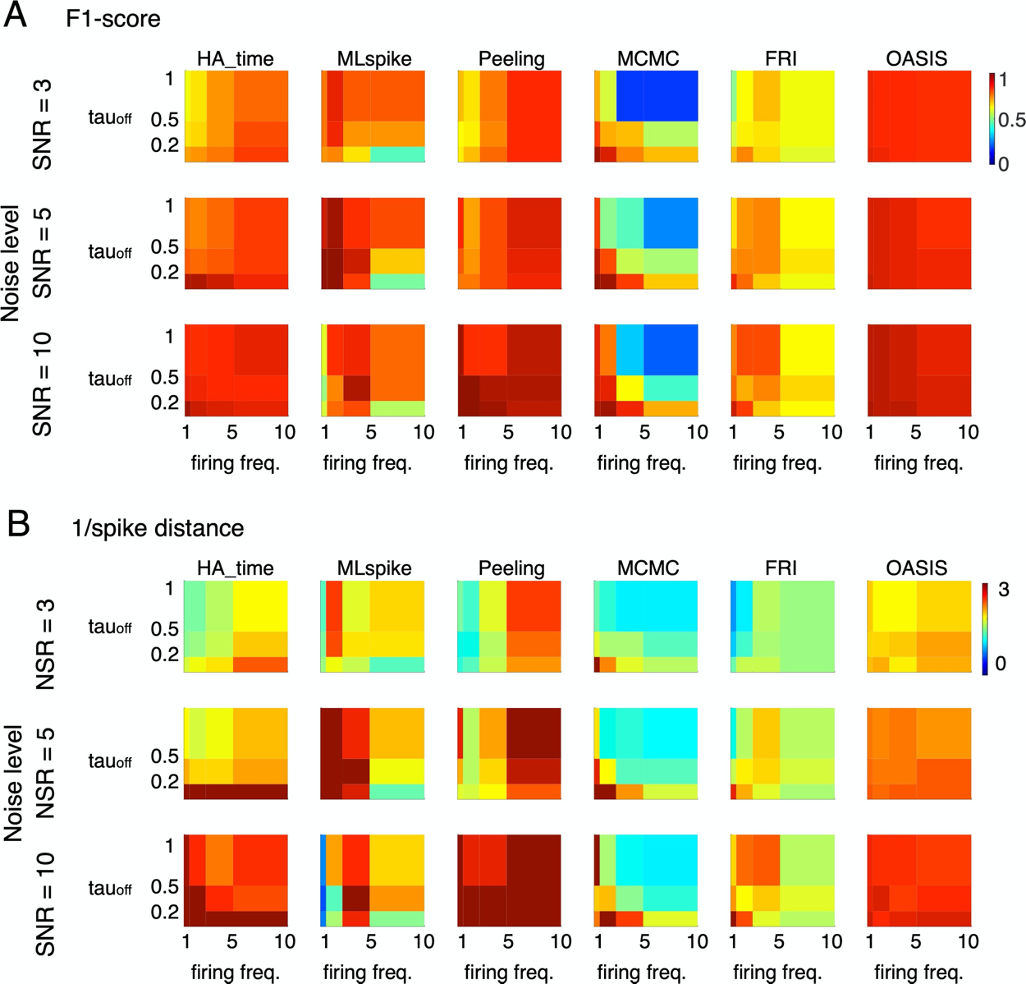


**Supplementary Figure S3: Performance of HA_time and benchmark algorithms for simulation data with variation in the SNR of Ca response signals.** **(A)** F1 score and **(B)** inverse of spike distance for three different SNRs (3, 5, 10) of the Ca response signals. The conventions are similar to Fig. S2.


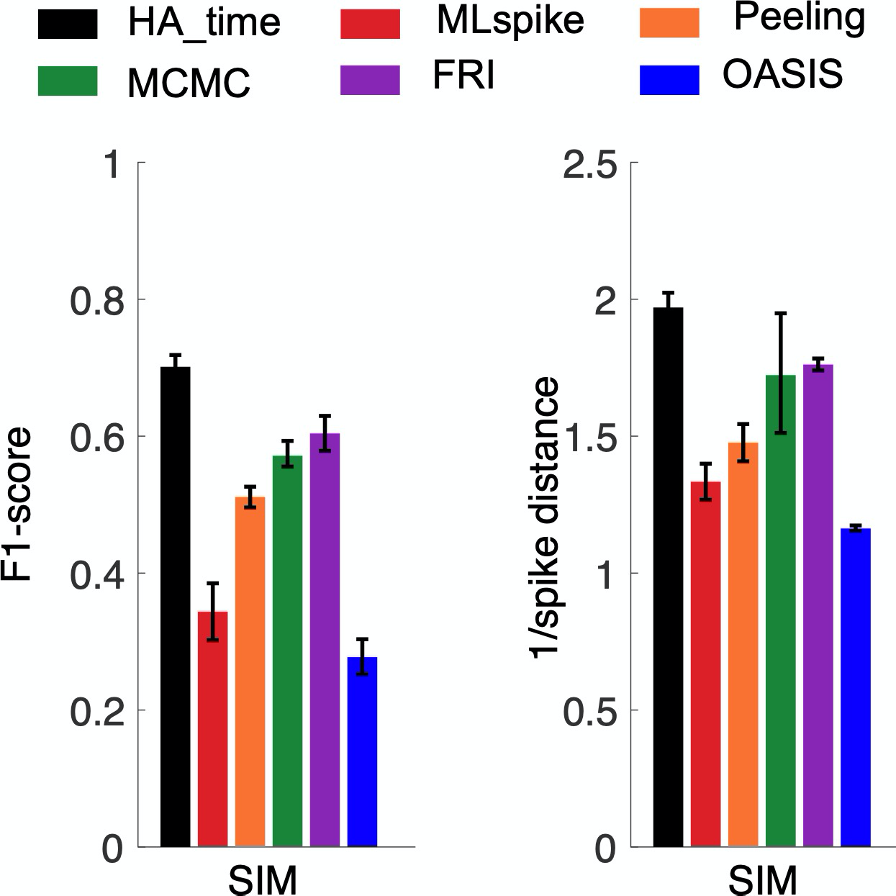


**Supplementary Figure S4: Performance benchmark of unsupervised HA_time and the unsupervised benchmark algorithms for the simulation data.** F1 score (left) and 1/spike distance (right) of HA_time and the benchmark algorithms for the simulation data (τ_1_, 0.01 s, τ_2_, 0.5 s, sampling rate, 10 Hz).


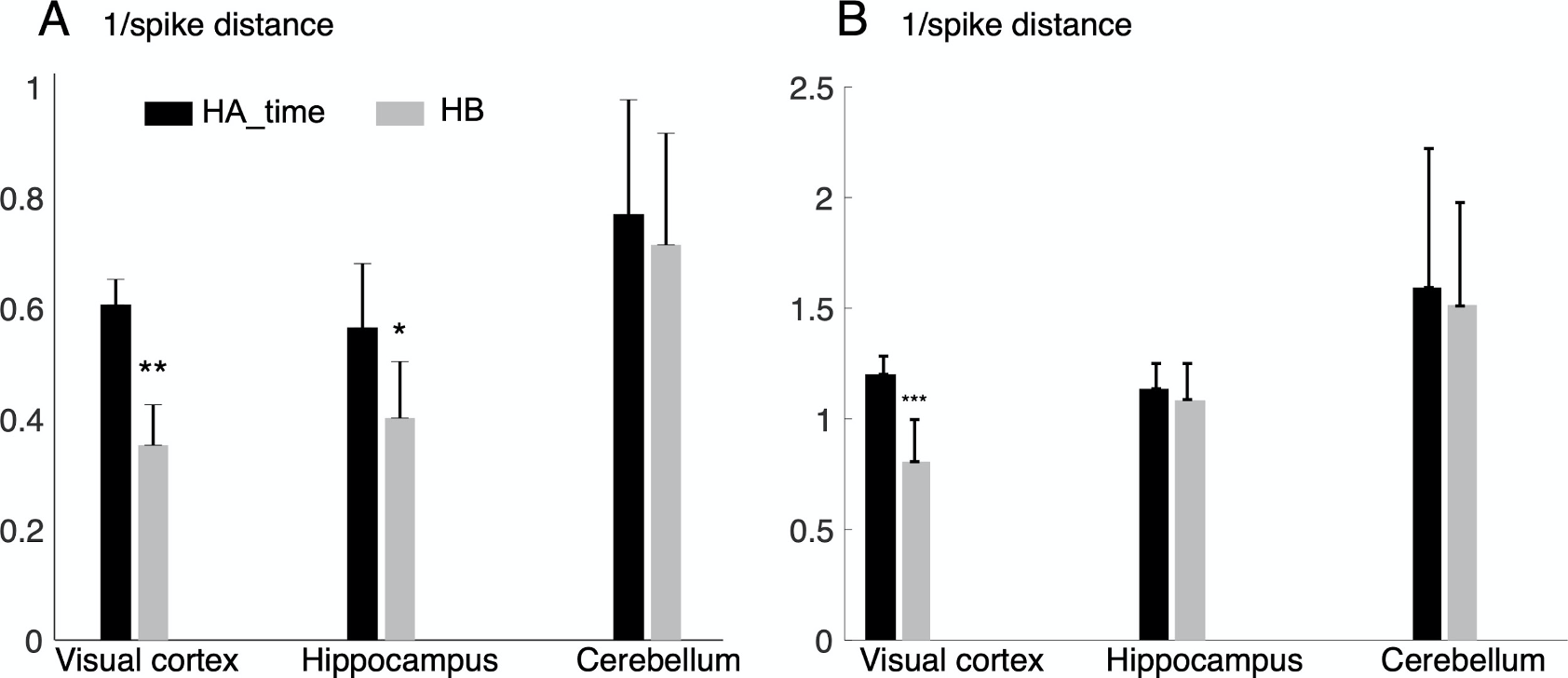


**Supplementary Figure S5: Performance benchmarks of HA_time and HB for the experimental data. (A)**F1 score and **(B)** inverse of the spike distance for HA_time (black columns) and HB (gray columns).


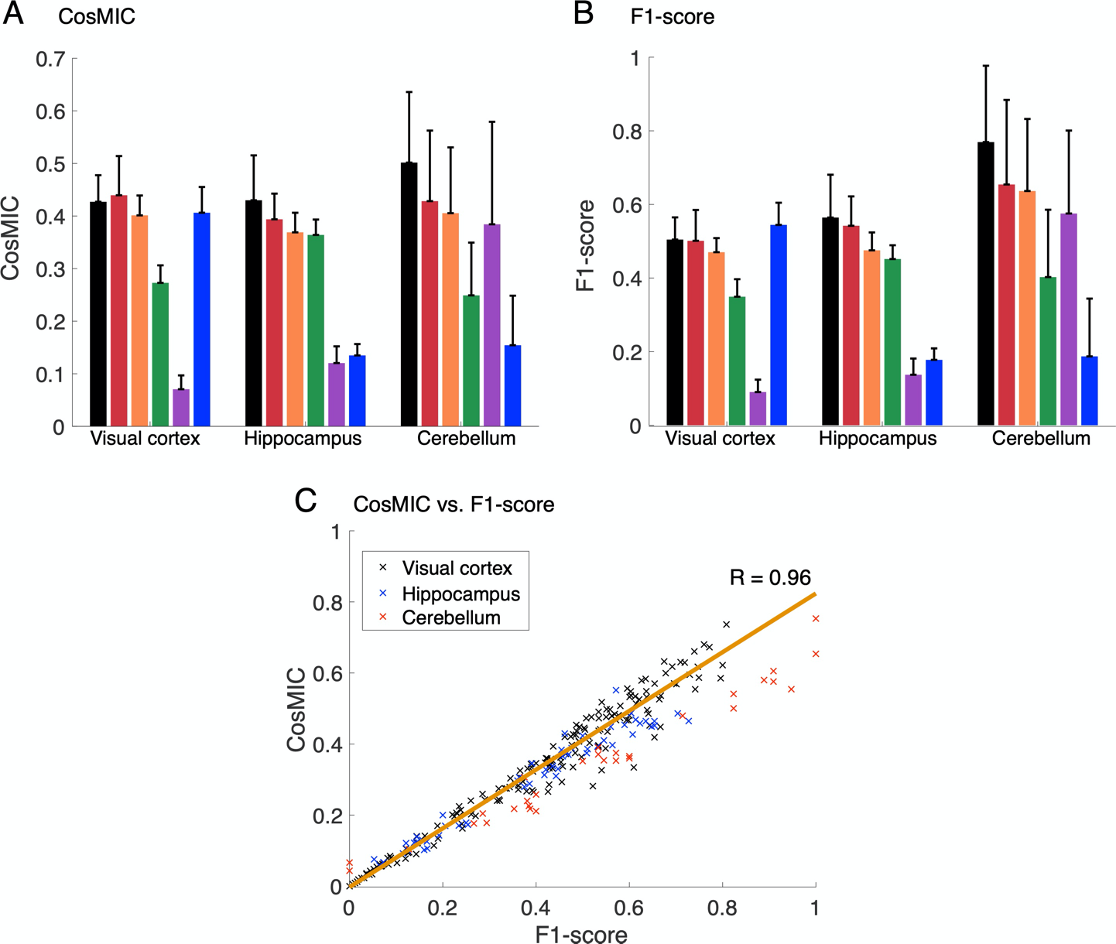


**Supplementary Figure S6: Correlation analysis of CosMIC and F1 score. (A)**CosMIC and **(B)** F1 score of HA_time and the benchmark algorithms for the three experimental data sets. Correlation analysis of CosMIC and F1 score **(C)** showed a strong correlation coefficient (r = 0.96). The conventions are the same as in Fig. 6.

| **#** | **Area** | **Brain state** | **Dye** | **n** | **Sampling rate (Hz)** | **Est. τ_1_**  **(ms)** | **Est. τ_2_**  **(ms)** | **Ref. τ_2_ (ms)** | **References** |
| --- | --- | --- | --- | --- | --- | --- | --- | --- | --- |
| 1 | V1 | AN | GCaMP6f | 11 | 60.1 | 10 | 200 | 200–400 | 35,39 |
| 2 | V1 | AN | GCaMP6s | 9 | 60.1 | 50 | 750 | 700–800 | 35,39 |
| 3 | V1 | AN | GCaMP5k | 9 | 50 | 100 | 500 | 500–700 | 35,41 |
| 4 | HP | Slice | OGB-1AM | 9 | 10 | 100 | 750 | 500–1000 | 16,34 |
| 5 | CB | AN | Cal-520 | 5 | 7.8 | 50 | 400 | 200–700 | 33 |

**Supplementary Table S1: Summary of the experimental data sets used in the performance benchmark.** The experimental data include simultaneous recordings from three different brain regions (visual cortex, hippocampus, and cerebellum) using a variety of Ca dyes. V1: visual cortex, HP: hippocampus, CB: cerebellum. AN: anesthetized. Est. τ_1_ and Est. τ_2_—rising and decay constants, respectively, estimated by HA_time. Ref. τ_2_—decay constant referenced from the literature.
